# The Beneficial Role of Polysaccharide Hydrocolloids in Meat Products: A Review

**DOI:** 10.3390/gels11010055

**Published:** 2025-01-10

**Authors:** Hanxiao Bao, Yuxi Wang, Yue Huang, Yuhao Zhang, Hongjie Dai

**Affiliations:** 1 College of Food Science, Southwest University, Chongqing 400715, China; baohanxiao@stu.scau.edu.cn (H.B.); wyx54088@email.swu.edu.cn (Y.W.); zhy1203@swu.edu.cn (Y.Z.); 2 College of Food Science, South China Agricultural University, Guangzhou 510642, China; 3 Chongqing Sericulture Science and Technology Research Institute, Chongqing 400700, China

**Keywords:** polysaccharide hydrocolloids, cellulose, starch, meat products, application

## Abstract

Polysaccharide hydrocolloids have garnered increasing attention from consumers, experts, and food processing industries due to their advantages of abundant resources, favorable thickening properties, emulsification stability, biocompatibility, biodegradability, and high acceptance as food additives. This review focuses on the application of polysaccharide hydrocolloids and their beneficial roles in meat products by focusing on several commonly used polysaccharides (i.e., cellulose, chitosan, starch, sodium alginate, pectin, and carrageenan). Firstly, the recent advancements of polysaccharide hydrocolloids used in meat products are briefly introduced, along with their structure and potential application prospects. Then, the beneficial roles of polysaccharide hydrocolloids in meat products are comprehensively summarized and highlighted, including retarding lipid and protein oxidation, enhancing nutritional properties, improving texture and color quality, providing antibacterial activity, monitoring freshness, acting as a cryoprotectant, improving printability, and ensuring security. Finally, the challenges and opportunities of polysaccharide hydrocolloids in meat products are also introduced.

## 1. Introduction

Polysaccharide hydrocolloids are colloidal systems that arise from the dispersion of polysaccharide molecules in aqueous media. The presences of hydrophilic groups, namely hydroxyl, carboxyl, amino and carboxylate on the polysaccharide molecules promote the formation of these hydrocolloidal systems through hydration [1]. Generally, polysaccharide hydrocolloids can be categorized into various types based on their sources, including algal polysaccharides (e.g., carrageenan and alginate), animal polysaccharides (e.g., chitin, hyaluronic acid), plant polysaccharides (e.g., cellulose, starch, konjac gum, and pectin), microbial polysaccharides (e.g., xanthan gum and gellan gum), and modified polysaccharides (e.g., carboxymethyl cellulose and modified starch) hydrocolloids. Recently, these polysaccharide hydrocolloids have shown diverse applications in the food industry, such as gel formation [2], emulsion stabilization [3], fat replacement [4], and packaging film or coating materials [5], due to their excellent gelling and film forming ability, emulsification and thickening properties, as well as swelling and water retention properties [6,7,8,9,10].

**Figure 1:**
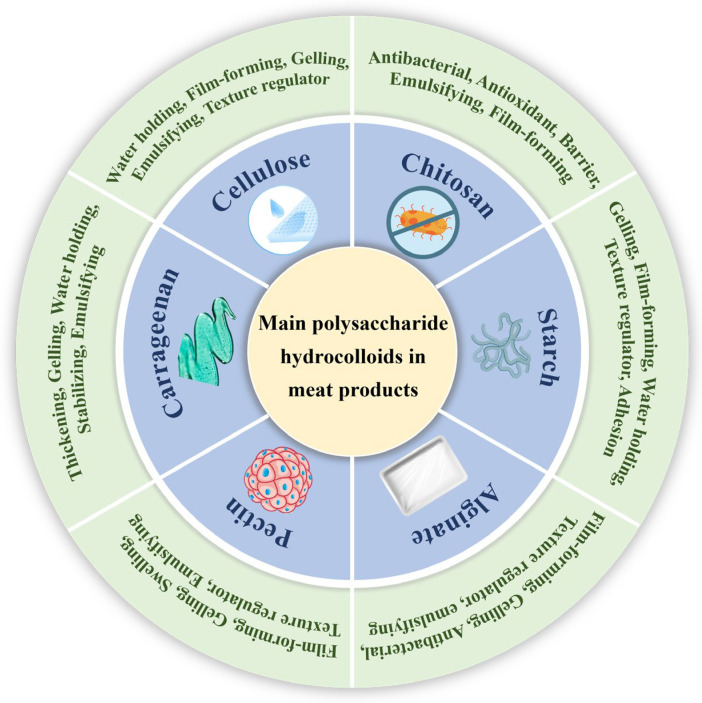
The several common polysaccharide hydrocolloids used in meat products and their main properties valid for its application.

Many forces between meat proteins such as disulfide bonds, hydrophobic interaction, electrostatic interaction, and hydrogen bonding can affect the quality of meat products [11]. Usually, polysaccharide hydrocolloids can interact with proteins (e.g., myofibrillar protein) through intermolecular forces including electrostatic attraction or repulsion, van der Waals, steric, hydrophobic, or hydrogen bonding [12]. These regulated interactions enable polysaccharide hydrocolloids to play a wide range of roles in meat product processing, such as improving the gelation and emulsification properties of meat protein [12,13]. Furthermore, the polysaccharide hydrocolloids process the typical characteristics of dietary fiber and prebiotics as well as specific physicochemical behavior, which can be used to ameliorate the nutritional and technological profile of meat products [7]. The oxidations of lipids and proteins as well as microbial reproductions have always threatened the quality and safety of meat products during slaughtering, processing, transportation, storage, and marketing [11]. The introduction of polysaccharide hydrocolloids can be effective in solving these threats [14,15]. For example, some polysaccharide hydrocolloids possess antioxidant or antimicrobial properties such as chitosan [6] and sodium alginate [16], thereby effectively preventing lipid and protein oxidation and delaying degradation of meat products [17,18]. Due to their excellent mechanical strength and flexibility, some polysaccharide hydrocolloids (e.g., chitosan, starch, cellulose, and pectin) can serve as matrix materials for coatings or films [19,20]. Polysaccharide hydrocolloids can also effectively prolong the shelf life of meat products by combining with active compounds [21,22] and enhance the texture and color of meat products, thereby improving their sensory quality [23]. In addition, polysaccharide hydrocolloids can be used to replace fat in meat products or to improve the 3D printing properties of meat products [24,25]. Benefiting from the development and research of polysaccharide hydrocolloids, a number of personalized meat products, such as low-fat and low-salt meat products, have been developed to cater to consumer preferences [4,14]. Several common polysaccharide hydrocolloids used in meat products and their main properties are shown in Figure 1.

Recently, the researchers and scientific community have paid attention to developing the applications of polysaccharide hydrocolloids in meat products. For example, introducing cellulose or chitosan can improve the gel properties of meat products [11,26], adding starch or carrageenan enables meat products to exhibit excellent water-holding capacity [27], and some polysaccharide hydrocolloids stabilized emulsion gels can be used as fat replacers in meat products [28,29]. Based on these published studies, some reviews regarding polysaccharide hydrocolloid application in meat products have been published, mainly focusing on the application of modified cellulose [11], chitosan [6], gums [30], and cereal polysaccharides [7] in meat products, and the use of natural polysaccharides as cryoprotectants and/or cryostabilizers for meat products [31]. Even though there are already some reviews involving the use of polysaccharide hydrocolloids in meat products, there are currently limited overall reviews available that comprehensively describe the beneficial roles and the overall potential application of various polysaccharide hydrocolloids in meat product processing and preservation purposes. In order to efficiently summarize the latest application of various types of polysaccharide hydrocolloids in meat products, we mainly gathered the recent advances that are related to this topic. This review aims to provide a summarization and analysis of several polysaccharide hydrocolloids (i.e., cellulose, chitosan, starch, sodium alginate, pectin, and carrageenan) commonly used in meat products. The beneficial roles of these hydrocolloids in meat products were summarized comprehensively for the first time, including retarding lipid and protein oxidation, enhancing nutritional properties, improving texture and color quality, providing antibacterial activity, monitoring freshness, acting as a cryoprotectant, enhancing printability, and ensuring security. This review is helpful to clarify the role of polysaccharide hydrocolloids in meat products and develop novel meat products.

## 2. Serval Common Polysaccharide Hydrocolloids Used in Meat Products

Polysaccharides hydrocolloids, having strong health attributes and unique physicochemical properties, can be applied to regulate the composition, nutrition, texture, and shelf-life as well as enrich the product form of meat products. Recently, several common polysaccharide hydrocolloids have been widely used in meat products including cellulose, chitosan, starch, sodium alginate, pectin, and carrageenan, which are introduced and discussed in the following part. Some representative examples involving the species, roles, and applications of polysaccharide hydrocolloids in meat products are summarized in Table 1.

### 2.1. Cellulose

**Figure 2:**
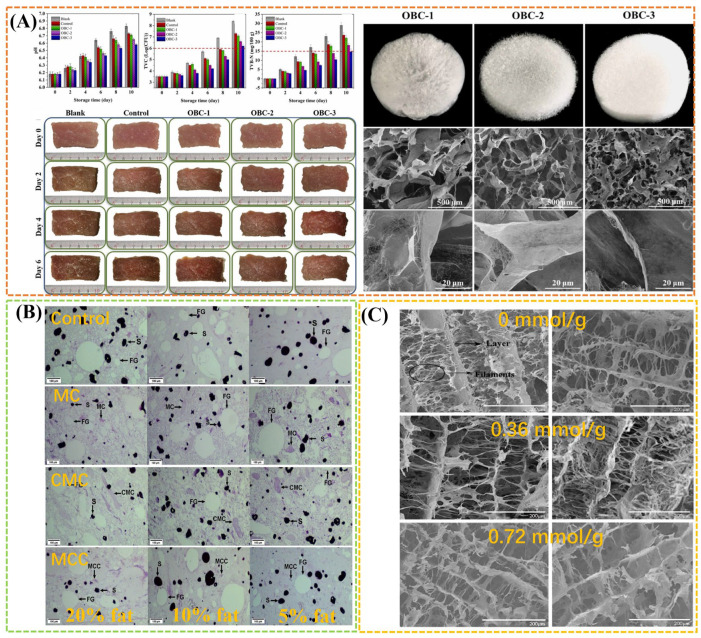
(**A**) Effect of OBC mats on the preservation of chilled meat, and the appearance and micromorphology of OBC mats. Reproduced with permission from [51]; (**B**) light micrographs of raw meat batters formulated at the targeted fat levels of 20%, 10%, and 5% and prepared without (Control) or with three modified cellulose ingredients (i.e., MC, CMC, and MCC). Reproduced with permission from [53]; (**C**) SEM images of cull cow myofibrillar protein gel containing various cCNF addition with different carboxyl content. Reproduced with permission from [54].

Cellulose, as the most abundant natural polymer on earth, is composed of β-D-glucopyranosyl groups linked by β-1,4 glycosidic bonds. Massive hydroxyl groups in the crystallization zone can form strong hydrogen bonds within and between molecules, which hinder the effective contact and interaction between the cellulose surface and other substances, mostly limiting its application in meat products [11]. Thus, modification of cellulose is an important strategy to expand the application of cellulose in the food industry. Various modification approaches of cellulose including physical, chemical, biological, and combined methods can be effectively employed to adjust the main characteristics such as water retention, solubility, size, morphology, surface charge, adsorption, gelling, and film forming ability [8,50], resulting in the intensive applications of microcrystalline cellulose (MCC), nanocellulose, carboxymethyl cellulose (CMC), methyl cellulose (MC), hydroxypropyl methyl cellulose (HPMC), and ethyl cellulose (EC) in meat products. Recently, the cellulose derivatives have been well applied to meat preservation as packaging materials (e.g., indicator film, packaging film, pad, and coating) [51,52]. A typical study indicated that TEMPO-oxidized bacterial nanocellulose (OBC) can be self-assembled into a preservation mat with antibacterial and high water absorption properties, showing a good effect on the preservation of chilled meat. These properties were associated favorably with the carboxyl content of OBC induced by TEMPO oxidation (Figure 2A) [51].

In addition, most of cellulose and cellulose derivatives can augment the dietary fiber content of meat products as a functional ingredient, or be used as plant-based meat analogs to replace traditional meat products based on their water retention, emulsification and gel properties [11,53]. For example, MC, CMC, and MCC as the modified cellulose ingredients have been applied as a fat replacer in reduced-fat meat batters with targeted fat levels of 20%, 10%, and 5%. Among them, MCC has the best overall effect due to its well distribution within the raw batter and similar levels of cooking loss and textural hardness compared the controls (Figure 2B) [53]. The addition of carboxymethylated cellulose nanofibril (CNF) significantly enhanced water-holding capacity and structure of cull cow meat myofibrillar protein gels, thus improving the juiciness and tenderness of meat products to raise the quality. This improvement effect is related to the degree of substitution of CNF carboxymethylation, which can determine the network structure of myofibrillar protein gels (Figure 2C) [54]. Cellulose-based film and hydrogel added with chitosan-citric complex showed excellent antibacterial effect on *Escherichia coli* (*E. coli*) and *Staphylococcus aureus* (*S. aureus*) and thus can be used as fresh meat packaging to extend the shelf life of meat products [55].

Generally, there are two approaches to add cellulose into meat products, namely the direct addition of cellulose and the indirect addition in the form of cellulose stabilized emulsion [11]. For example, cellulose could regulate nutrient composition and additive dosage in meat products by playing the role of polyphosphates and fat substitute. The authors of [33] found that the incorporation of 0.5% CNF in conventional formulations of emulsion-based sausages can effectively substitute 0.5% polyphosphates or 1% starch, showing similar quality characteristics of sausages. The CNF gels exhibited good stabilizing properties and acted as water-binding agents in emulsion-based meat products. Moreover, CNF and CNF-stabilized palm oil Pickering emulsion could act as fat substitutes in the field of emulsified sausages processing. Emulsified sausage with 30% pork back fat being replaced possesses better quality and texture, such as lower fat content, higher moisture content, and the hardness, chewiness, and springiness as well as sensory score could also be enhanced compared with the full-fat control one [56]. Furthermore, the functional groups of cellulose-based edible coatings can react with the precursors of acrylamide and heterocyclic amines during the frying process, thereby effectively weakening the generation of these carcinogens. Considering the effect on carcinogens, cellulose-based edible coatings have a positive application value in improving the quality and safety of fried and grilled meat products [57]. However, unlike the natural cellulose, the chemical groups or morphology of the modified cellulose and nanocellulose are changed, thus their safety and nutritional qualities should be of concern after digestion when they are applied in meat products in the form of direct addition [11]. Furthermore, the polyhydroxyl and easily modified properties of cellulose make it easy to introduce more functional ingredients into meat products containing cellulose. Overall, as a packaging material and ingredient in direct contact with meat, the effect and interaction mechanism between cellulose and nutrient components such as protein, fat, and flavor in meat products still needs to be systematically studied, especially the charged cellulose. Furthermore, the safety of nanocellulose should be considered when they are applied in meat products.

### 2.2. Starch

Starch is abundantly present in cereals, legumes, as well as roots and tubers of plants [58]. Amylose and amylopectin are two forms of starch molecules, having a simple linear and a more complex branched structure. Moreover, amylose is connected by α-1,4 glycosidic bonds, while amylopectin is connected by α-1,6 and α-1,4 glycosidic bonds [59]. The starch with high amylose content displays higher gel strength, while the starch with more amylopectin fractions can form more viscous, elastic gel and is easier to cook and easily gelatinizes at low temperature [7]. At present, the starches available are physically and/or chemically modified in addition to genetic and enzymatic modifications. Generally, the modified starch is widely used in the food industry because of its enhanced functional and gelatinization properties compared to the native starch [7]. Furthermore, the modified starch can provide a more beneficial effect on improving the water retention of meat products than that of native starch, thus also providing better adhesion performance to bond meat filling and mince together [27]. The starches with the same granular size like fat emulsions can be designed as fat replacers in low-fat foods due to their high absorbing and binding ability to water molecules [7]. An oil-modified crosslinked starch has been shown to serve as a viable fat substitute in meat products on account of its positive effect on the protein gel network, water retention of pork meat, and diminishing fat and saturated fatty acid levels additionally [27]. Additionally, the research of Eshag Osman et al. [42] suggested that acetylated corn starch can be utilized as a fat substitute in meat products by strengthening the physicochemical and sensory quality of beef patties.

**Figure 3:**
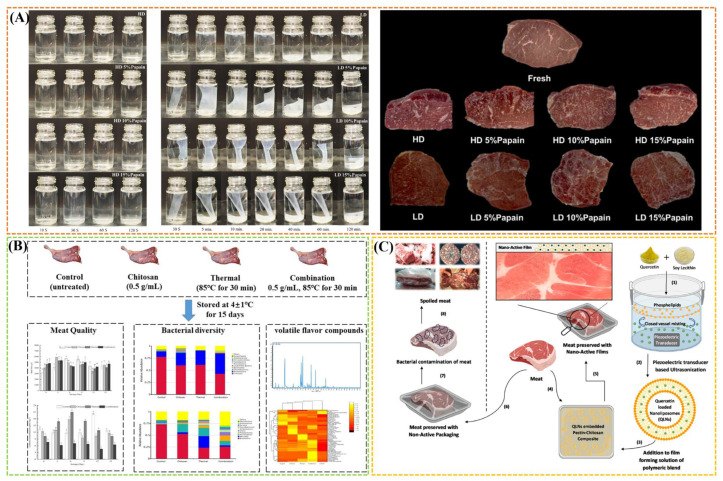
(**A**) Water solubility as a function of time in edible starch films with high dissolution (HD) and low dissolution (LD) containing various papain concentrations (5–15%), and the appearance of packaged meat in films after incubation for 30 min. Reproduced with permission from [39]; (**B**) effect of chitosan coating on physicochemical properties, bacterial diversity, and volatile flavor of braised duck meat during refrigerated storage. Reproduced with permission from [67]; (**C**) schematic representation of quercetin loaded nanoliposomes embedded in pectin-chitosan based packaging films against meat-associated spoilage pathogens. Reproduced with permission from [68].

Notably, starch is the most popular type of polysaccharide used in edible film. Starch-based films with higher amylose content generally display better mechanical and gas barrier properties. It is because amylopectin retrogrades more slowly than amylose due to the branched nature of the amylopectin molecule [59]. Recently, the electrospinning method emerged as a versatile and viable approach, facilitating the use of natural biopolymers (e.g., polysaccharide hydrocolloids) to produce biodegradable packaging [60]. Generally, starch with high-amylose starches (>50% of amylose content) are being recommended for electrospun nanofiber production [61,62]. Furthermore, electrospun starch nanofibers have a high capacity, stabilization, and controlled release effects for active agents, which can improve the overall quality and increase the shelf life of meat products [63]. Starch can also be effectively combined with hydrophobic polymers to fabricate double-layer film packaging. This strategy can effectively block the entry of oxygen and water, thereby prolonging the shelf life and preserving the freshness of pork [64]. Starch can be made into antibacterial packaging films by introducing different bioactives (e.g., gallic acid, chitosan, and carvacrol), which can inhibit the growth of *L. monocytogenes* in meat products [65]. Moreover, introducing starch can improve the 3D-printed properties of meat products, enhancing its fluidity in the extrusion process and the quality [66]. As alternative ingredients that satisfy emerging consumers demand for clean label product, native starch also have intensive potential application regarding the development for functional properties and textural and eating quality of meat products. Enhanced levels of corn starch incorporation as a fat replacer resulted in a rising trend in the water binding capacity of meat sausages due to its ability to absorb and bind water [38]. It is well known that incorporating functional ingredients into edible films can improve the texture, color, and flavor of meat products. Wongphan et al. [39] fabricated a novel functional edible starch film with incorporated papain for meat tenderization. The edible films were characterized at varying papain concentrations (5–15%) using high dissolution (HD) and low dissolution (LD) cassava starch with different water solubilities. The films containing higher papain content facilitated tenderization, while the dissolution of films accelerated the release and tenderization. Furthermore, the papain-incorporated films showed a deeper bright red color due to the enzymatic catalysis influencing the transformation and chemistry of myoglobin (Figure 3A). Overall, starch takes different roles in meat products. Starch with abundant, safe, and inexpensive characteristics shows good potential for commercial use to replace plastic filming or coatings. Moreover, it can act as a functional ingredient or filler to improve textural properties or to bind water as fat substitutes. In the application of meat products, the interaction between meat protein and starch with different sources and types would be the focus of the following research.

### 2.3. Chitosan

Chitosan is a kind of polysaccharide derived from the deacetylation of chitin, which exists widely in nature as the second most abundant polysaccharide after cellulose [69]. The structure of both chitin and chitosan is highly similar to that of cellulose which consists of β-(1–4) linked d-glucose units, as a naturally occurring cationic polysaccharide, chitosan has excellent physicochemical properties, such as antibacterial and antioxidant capacity and film-forming ability [6]. For example, Arkoun et al. [70] found that the electrospun chitosan-based nanofibers can effectively extend the shelf life of fresh meat by one week. Recently, the electrospun chitosan nanofiber-based packaging has garnered increasing attention due to superior functionalities [71]. Cui et al. [72] fabricated chitosan electrospun nanofibers containing tea tree oil liposomes, which exhibited high antibacterial activity against *Salmonella* and had almost no impact on the sensory properties of chicken meat, effectively keeping the food quality of chicken meat after 4 days of storage at 4 °C and 12 °C. Furthermore, chitosan has been recognized as GRAS (generally recognized as safe) as a food additive, dietary fiber, and functional ingredient by the US Food and Drug Administration. Based on these superior properties, chitosan is a potential edible packaging film or coating material and can also be used as a direct additive in meat products. Recently, the published studies have confirmed the positive effects of pristine chitosan in meat products [35,67] and the improved effects by introducing functional ingredients such essential oils, polymers, and nanoparticles on its mechanical and barrier properties as well as antimicrobial and antioxidant properties [68,73]. For examples, Li et al. [67] confirmed that combining chitosan coating with thermal treatment can effectively reduce carbonyl concentrations and inhibit total viable counts, *Enterobacteriaceae* counts, and the proportion of four primary spoilage organisms and produce the most volatile flavor compounds, which can be applied as an effective preservation method to improve the quality and prolong shelf life of braised duck meat (Figure 3B).

In another study, quercetin-loaded nanoliposomes can be effectively incorporated into the chitosan film, thereby endowing the film with excellent antimicrobial potential against multidrug-resistant meat pathogens bacterial contamination to extend the shelf life of meat products (Figure 3C) [68]. In addition, chitosan coatings have benefits in improving the quality of meat products such as decreasing moisture loss and delaying lipid oxidation and discoloration [6]. Duran and Kahve [74] found that the chitosan coating on beef reduced the total mesophilic aerobic bacteria (TMAB), lactic acid bacteria (LAB), and total volatile basic nitrogen (TVB-N) values and achieved a complete inhibition of *Stapylococcus aureus* (*S. aureus*) up to day 15 of storage. Similarly, the edible chitosan coating on beef and mutton cuts were beneficial in controlling the growth of *S. aureus* and *Listeria monocytogenes* (*L. monocytogenes*) [35]. Xiao et al. [18] developed an antibacterial chitosan/pullulan edible film loaded with carvacrol. This film demonstrated excellent antibacterial activity against various common bacteria found in chilled meat, such as *Pseudomonas fluorescens* (*P. fluorescens*), *L. monocytogenes*, *S. aureus,* and *E. coli*. The film effectively extended the shelf life of chilled goat meat to over 15 d. Furthermore, Ozaki et al. [36] found the positive effect of direct chitosan addition into the fermented cooked sausage regarding pH, color, sensory, and microbiological attributes, showing potential as a substitute for synthetic nitrite. Overall, chitosan used in the form of ingredient and films/coatings improves the quality and the shelf-life of meat products. Notably, chitosan films are soluble in aqueous medium with low concentration of organic acid such as acetic acid and even interact easily with water, showing poor physicochemical properties below the level of industrial packaging. Thus, as an antibacterial packaging material used in meat products, more research should be focused on the improvement of the physicochemical properties of chitosan films.

### 2.4. Sodium Alginate

Sodium alginate, as a salt of alginic acid and linear copolymers of α-(1 → 4) linked L-guluronic acid and β-(1 → 4) linked D-mannuronic acid, is a natural linear anionic polysaccharide isolated from the cell walls of brown algae [75]. Especially, sodium alginate also has unique gelling ability due to its strong ability to cross-link with Ca^2+^ to form strong gels and insoluble polymers, which can be used as ideal food coatings [76]. In general, the roles of sodium alginate in meat products can be broadly categorized into three types as follows: one is as an edible film or coating material [16,76], the other is as a fat substitute in meat products [77,78,79], and the third is to improve the sensory and nutritional of meat products as food ingredients [80,81]. In detail, as a film-forming or coating material, sodium alginate solutions are achieved by reducing the pH value or ionic complexation, resulting in the formation of a robust film or coating with a gel or fiber structure [16,76,82].

Furthermore, sodium alginate can serve as a substitute for fat in the production of nutritious meat products, exemplified by its application in the creation of low-fat frankfurters as a replacement for pork backfat, which improved the protein structure of frankfurters and reduced the fat and energy [78]. In addition, using sodium alginate and konjac flour to prepare an emulsion hydrogel and enriched with encapsulated oil with flaxseed flour, could be used as a fat substitute in beef burgers [77]. Moreover, sodium alginate edible coating added with basil (*Ocimum* spp.) endows the beef with more attractive color performance [83]. The calcium–alginate-containing *Artemisia fragrance* essential oils as a potential antioxidant and antimicrobial coating improves quality stability and prolongs the shelf life of chicken breast meat without a negative impact on organoleptic properties [76]. In general, the current research on the application of sodium alginate in meat products is still focused on the field of meat preservation such as packaging filming and coating. In fact, sodium alginate can be designed as emulsifier, gelling, thickening agents in the restructured meat products, which can improve the juiciness, texture, color, and flavor of meat products. More research could be considered in 3D-printed and reconstituted meat products.

### 2.5. Pectin

Pectin is a heteropolysaccharide with three domains constituting its fine structure, including homogalacturonan, rhamnogalacturonan-I, and rhamnogalacturonan-II, which is a functional food ingredient as a hydrocolloid gum [84]. Commonly, pectin is widely used as packaging films or coatings for meat preservation or binder in meat products [85,86]. Generally, pectin with a degree of methyl-esterification higher than 50% or lower than 50% is categorized as high-methoxyl pectin (HMP) or low-methoxyl pectin (LMP). Notably, the HMP films usually display a higher elastic modulus than that of LMP films [87]. Moreover, pectin displays great potential to maintain the emulsion stability of meat batters [88]. Thus, pectin can be used to mimic the physical and sensory properties of emulsified fats since the consistency and deformability of pectin hydrogel particles are similar to the fat particles.

For example, the food clean-label binders prepared by sugar beet pectin, pea protein, laccase, and methylcellulose hydrogel were used to combine protein with fat to produce bacon [88]. Zheng et al. [89] used the amino acid-modified pectin as a fat replacer in minced chicken breast, which exhibited excellent water retention capability. After thermal processing, there was no significant difference in chroma and texture and the electronic tongue sensory evaluation between the amidated pectin group and the control group. Furthermore, the amidated pectin can offset the attenuation of muscle protein crosslinking capacity in low-salt chicken produce and enhance the hardness of samples. Moreover, the utilization of pectin-based packaging, incorporating plant extracts, has demonstrated a food preservation effect due to its specific antibacterial and antioxidant properties [84]. Sani et al. [90] fabricated a complex film composed of potato starch and apple peel pectin incorporated with microencapsulated *Zataria multiflora* essential oil and zirconium oxide nanoparticles. This composite film had certain antibacterial and antioxidant capacities to prolong the shelf life of quail meat as an active packaging material. Overall, the current research of pectin used in meat products mainly focuses on the field of films or coatings. As a natural adhesive agent, the potential application of pectin in meat analogues, fat substitutes, and 3D-printed meat products are worth further exploration.

### 2.6. Carrageenan

Carrageenan, a hydrophilic polysaccharide sulfate colloid extracted from red algae, consists of galactose and dehydrated galactose. According to the binding forms of sulfate, carrageenan can be divided into seven types in total: κ-, ι-, λ-, γ-, ν-, ξ-, and μ-types, with the first three (κ-, ι-, and λ-carrageenan) being the most prevalent in the food industry, which contain D-galactose repeating units and one, two, or three of negatively charged organosulfates, respectively. Moreover, the degree of sulfate substitution determines the properties of carrageenan such as water holding, thickening, gelling, and stabilizing [9]. Due to its superior physiochemical properties, such as cold solubility, freeze–thaw capability, gelling property, and hydrophilicity, carrageenan exhibits extensive application as a food additive in the meat products as an efficient thickening, stabilizer, emulsifying or gelling agent, which can effectively increase the water and fat binding capacity, gel properties, and textural profiles of meat products [9,49].

Especially, adding carrageenan to meat products during production can reinforce its rheological and water retention abilities. Notably, both the addition forms (dry powder and pre-suspended in water) of carrageenan can improve the textural and gel properties of frankfurters and the rheological behavior of meat batter during heating. However, the pre-suspended carrageenan can form a denser and finer gel network due to its better dispersion [49]. Moreover, replacing part of the animal fat with carrageenan in low-fat meat products is beneficial to maintain a uniform texture [9]. The addition of 0.2% (*w*/*w*) κ-carrageenan to frankfurters resulted in an obvious enhancement in the texture and gel characteristics and improved the rheological properties of the meat batter. The fully pre-hydrated κ-carrageenan in water can be more uniformly dispersed in the meat protein gel matrix, thus improving the formation of hydrogen and disulfide bonds and generating a three-dimensional gel network [49]. Another similar study demonstrated that the addition of κ-carrageenan and transglutaminase can simultaneously improve gel performance and viscoelasticity of meat batter [48]. Shin et al. [91] found that the use of κ-carrageenan and duck fat instead of part beef fat and pork back fat in frankfurter not only kept its sensory qualities, but also strengthened its physicochemical properties and oxidative stability. Overall, carrageenan contributes to improved gel formation and water retention in meat products, especially in low-fat meat products. However, the interaction mechanism of carrageenan with meat proteins and the other meat components is not fully understood.

## 3. Beneficial Roles of Polysaccharide Hydrocolloids in Meat Products

**Figure 4:**
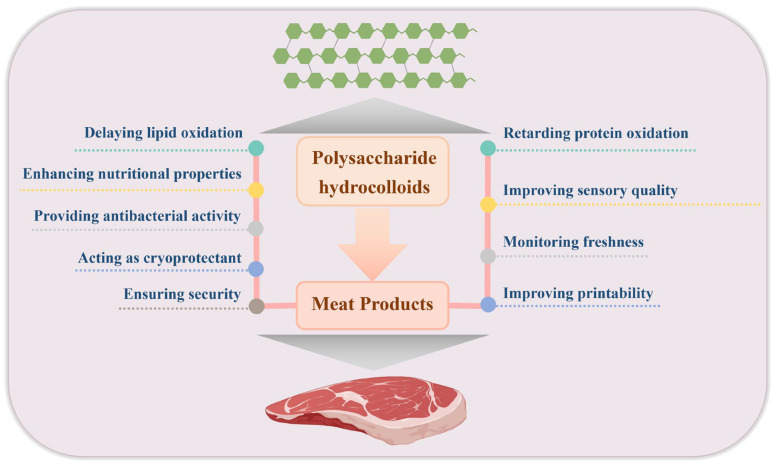
The positive roles of polysaccharide hydrocolloids in meat products.

As the difference and diversity in origin, composition, structure, and properties of various polysaccharide hydrocolloids, these polymers generally exhibit different functionalities when used in meat products. The following section mainly introduces the beneficial roles of polysaccharide hydrocolloids in meat products, which is summarized and depicted in Figure 4. Some representative examples involving the benefits of polysaccharide hydrocolloids in meat products are displayed in Table 2.

### 3.1. Delaying Lipid Oxidation

Lipid oxidation is recognized as the primary non-microbial factor contributing to the deterioration of meat products, leading to undesirable alterations in texture, color, smell, and overall quality, thereby impacting their nutritional value and the choice of consumers [92]. There are three different mechanisms that can explain lipid oxidation, including autoxidation, photo-oxidation, and enzyme-catalyzed oxidation. Autoxidation is the most vital form of lipid oxidation in meat products, which results in the generation of toxic substances accompanied by varying degrees of texture, odor, and color deterioration [93]. Moreover, the photo-oxidation produces hydroperoxides in the existence of light and sensitizers such as hemoglobin and myoglobin [92], and enzyme-catalyzed oxidation mainly induced by lipoxygenase can directly oxygenate polyunsaturated fatty acids to form lipid hydroperoxides in many different processed meat products [94]. Although lipid oxidation contributes to the generation of pleasant aromas, especially during ripening or dry-cured stages of meat products, it also can cause the quality deterioration of meat and meat products, ultimately shortening the shelf-life and endangering human health [17,92,94].

Recently, the utilization of polysaccharide hydrocolloids as antioxidants has become a novel approach to delay lipid oxidation during processing and storage of meat products. Two methods have been studied extensively, namely the direct addition of polysaccharide hydrocolloids (e.g., chitosan and pectin) as natural food additives and active ingredients or forming a coating film with antioxidant properties [17,26,64,94]. Chitosan exhibits antioxidant capacity by virtue of its ability to chelate with metal ions, which can trigger lipid oxidation [74]. Chaudhary et al. [95] proved that the free radicals, such as superoxide, hydroxyl, and alkyl, can be eliminated by chitosan-based emulsions due to its ability to serve as hydrogen donors to hinder oxidative sequence. Cellulose nanocrystals possess a low oxygen permeability under high relative humidity, rendering them suitable for enhancing the oxygen barrier capacities of other biopolymers when used in combination, thereby impeding lipid oxidation [17]. Moreover, pectin-based films or coatings show a reduced permeability to oils, fats, and oxygen, thus effectively retarding lipid oxidation [96]. Han et al. [97] found that the direct addition of chitosan and carboxymethyl cellulose (CMC) separately to minced pork meat resulted in evident alterations of protein–lipid network. CMC exhibited robust emulsifying properties at the protein–lipid interface, thus facilitating the incorporation of minute fat particles into the protein network to enhance the uniformity of the product. Notably, chitosan obviously promoted the aggregation of fat droplets and diminished the surface area of fat droplets, consequently resulting in a two-fold reduction of lipid oxidation products, whereas CMC had no significant effect on oxidation. El Sheikha et al. [98] investigated the effects of CMC coating concentration (0, 1, 2, 3, and 4%) of ethanolic propolis extract (EPE) on sustaining the quality and prolonging the shelf life of refrigerated chicken breast meat. The Thiobarbituric Acid Reactive Substances (TBARS) values of the samples coated with CMC and EPE at 2%, 3%, and 4% levels decreased while comparing with the samples uncoated or coated with CMC only. In general, due to their excellent gelation and film-forming properties, polysaccharide hydrocolloids can be used as edible carriers of active substances to delay lipid oxidation in meat products, either as direct additions or as packaging and coating materials.

### 3.2. Retarding Protein Oxidation

Protein oxidation, a process involving oxidative attack caused by reactive oxygen and modifications of side chain amino acids, leads to protein aggregation, carbonylation, and change of surface hydrophobicity [99]. A significant signal of protein oxidation is the generation of disulfides and carbonyl compounds, which gradually increases as oxidation proceeds. In the case of meat products, protein oxidation can exert a negative impact on both nutritional and sensory quality, mainly embodied in the loss of essential amino acids, altering digestibility and deterioration of flavor and aroma. Furthermore, protein oxidation in meat products exists at all stages, namely from slaughter to processing, storage, and especially in the high temperature processing treatment [100].

The direct addition of antioxidants or the preparation of antioxidant coatings are two crucial methods to impede protein oxidation in meat products. As to the direct addition of antioxidants, Song et al. [101] prepared microencapsulated procyanidins by extruding starch (MPS) and utilized MPS as a natural antioxidant to postpone protein oxidation and improve the quality of refrigerated chicken sausages. An obvious inhibitory effect on generation of disulfide bonds (only 46.76 μmol/g protein) appeared with the application of MPS in chicken sausages, which significantly retarded protein oxidation and maintained the texture. Mousa [102] found that the carbonyl protein values decreased while adding the composites of polysaccharide hydrocolloids (gum Arabic and CMC) and fruit peel flours acted as fat substitutes of hamburger, showing the reduction of protein oxidation during frozen storage. Chitosan nanoemulsion coating combined with the essential oils of two aromatic plants covering the turkey meat has been proven to hinder protein oxidation, showing a reduction of the carbonyl content [103]. Nevertheless, the utilization of antioxidants necessitates discreet consideration of the concentration and dosage. As one study showed, gallic acid/chitosan coating effectively suppressed myoglobin and lipid oxidation of fresh pork. However, it was noted that beyond a specific concentration threshold (0.4% gallic acid in 2% chitosan), the coating exhibited pro-oxidant properties on account of the high content of gallic acid [21]. Notably, most of the commercial polysaccharide hydrocolloids currently used in meat products do not have antioxidant properties, its function in antioxidants mainly depends on the interaction with active substances.

### 3.3. Acting as Cryoprotectant

**Figure 5:**
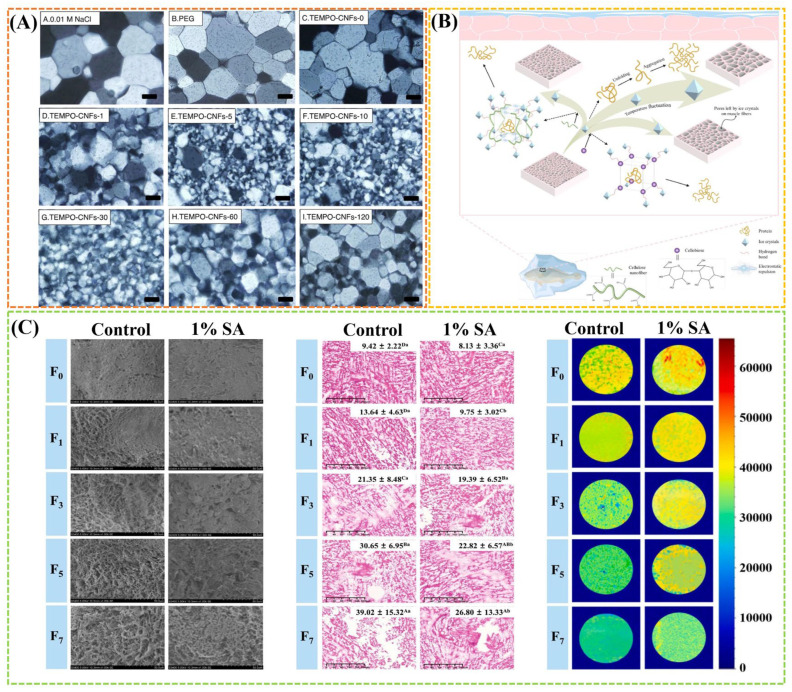
(**A**) Polarized light microscopy images of ice crystal wafers after 30 min annealing at −8 °C: 0.01 M NaCl, PEG (negative control), and TEMPO-CNFs sonicated for 0–120 min. Reproduced with permission from [104]; (**B**) schematic model of the mechanism for the effects of CB and CNF on protein denaturation during temperature fluctuation cycles. Reproduced with permission from [106]; (**C**) effect of sodium alginate ice glazing on the SEM images, optical microscope images, and proton density weighted images of the freeze-thawed fish balls during F-T cycles. Reproduced with permission from [107].

Meat products are often frozen, a widely used method of food preservation, to extend the shelf life. However, this process may disrupt muscle protein, further promoting protein denaturation, which leads to varying degrees of deterioration in terms of texture, flavor, and other sensory attributes. The utilization of cryoprotectants has proven to be an effective strategy in safeguarding food against freeze-induced damage. Some polysaccharide hydrocolloids, such as cellulose [104,105,106], carrageenan [107], starch [108], and pectin [31], exhibit notable cryoprotective properties in the area of meat products storage by impeding the growth of ice crystals or imparting cryo-stabilizing effects. Recently, nanocellulose as a cryoprotectant has captured the eyes of researchers. Li et al. [104] found that the ice recrystallization inhibition (IRI) activity of nanocellulose depends on their fibril length, and longer nanocelluloses are more IRI active that can obviously decrease ice crystal size (Figure 5A). The IRI activity of nanocellulose has also been confirmed in the frozen large yellow croaker fillets during temperature fluctuation cycle [106] and the *Nemipterus virgatus* surimi during frozen storage [105]. The abundant hydrogen bonds and large specific surface area of nanocellulose facilitate more binding sites for water and protein molecules. An increase in CNF concentration could effectively inhibit the growth of ice crystals and protect the protein stability (Figure 5B) [106]. Furthermore, introducing nanocellulose can delay decrease of salt soluble protein content and Ca^2+^-ATPase activity, and maintain the structural integrity of myofibrillar protein. Moreover, the gel strength of surimi gel improved due to nanocellulose, showing a more compact microstructure with denser surfaces [105].

The waxy starch cryogel was diffused in pork meat samples by high-intensity ultrasound, which could effectively mitigate structural damage of pork during cryopreservation. The size of the myofibrillar spaces lowered by nearly 75% due to the competition among free water, cryogel, and meat proteins [108]. Furthermore, some antioxidant polysaccharides can offer cryoprotection by inhibiting oxidative reactions of meat products through scavenging free radical or chelating metal ions [105,109]. In another study, the utilization of carboxymethyl chitosan (CMCH) as a cryoprotectant for pork patties was investigated [109]. The results demonstrated that CMCH could effectively alleviate the oxidative denaturation of myofibrillar proteins (MPs) in pork patties, and gel networks became more uniform and organized with less voids after storage as the increase of CMCH incorporation content, thereby preserving the structural integrity of MPs and enhancing the freezing characteristics of frozen pork patties. Li et al. [107] found that 1.0% (*w*/*v*) sodium alginate (SA) glazing improved the surface frost formation and the quality of frozen fish balls in repeated freeze–thaw (F-T) cycles (Figure 5C). After seven F-T cycles, the ice production and thawing loss decreased by 28.30% and 21.02%, while the whiteness increased by 10.40%. The microstructure of fish balls was smoother and more uniform with smaller ice crystal diameter. Besides conventional cryoprotection via inhibiting the formation of ice crystals, the polysaccharides hydrocolloids also can also stabilize food texture and generate cryo-stabilized effects on food components due to their diverse rheological properties and interactions with texture-contributed components [31]. Thus, the polysaccharide hydrocolloids can offer protection against freezing/thawing-induced drip loss due to their high water binding abilities, which is also beneficial for textural maintenance of meat products with high moisture contents [10].

### 3.4. Improving Sensory Quality

The texture of meat products plays a vital role in meat quality, and it can be regulated by modifying tenderness, gel properties, and protein networks after addition of polysaccharide hydrocolloids as ingredients [11,26,42]. Cellulose and its derivatives that possess good dispersibility, uniform nano-size, and abundant surface area are ideal for utilizing in texture modulation of meat products [110]. In an experimental study related to beef patties designed for individuals suffered from dysphagia, a combination of 0.895% CMC and 0.018% tapioca starch was incorporated into the patties to successfully enhance the texture and create soft and bite-sized products [110]. Piao et al. [111] studied the quality peculiarities of surimi gel. As a result, adding a remarkably low amount of TEMPO-oxidized cellulose nanofibrils (0.1 g/100 g surimi) achieved significant improvement of surimi gel networks, resulting in the obvious promotion of better surimi texture, such as gumminess, hardness, and chewiness. The structural changes of surimi gels could be attributed to the factor of more uniform, better connected, and stronger chemical bonds of the gel network induced by the addition of TEMPO-oxidized cellulose nanofibrils. Another study confirmed the positive effects of ultrasound with potassium alginate marination (UPA) on tenderizing old chicken breast meat (raised for 300 days), showing the decreased moisture loss and shear force. Notably, the ultrasound treatment facilitated the penetration of potassium alginate into muscles through capillary force and increased the space between fibers, thereby avoiding tissue shrinkage and excessive dehydration upon heating [81]. Especially, the addition of polysaccharide hydrocolloids (e.g., CMC, chitosan, and pectin) can alleviate the deteriorated textural properties and water-binding capacity that may occur in low-fat meat products [89,97]. Furthermore, Cao et al. [49] found that both κ-carrageenan powder or κ-carrageenan water suspension addition can endow frankfurters with higher hardness, adhesiveness, springiness, chewiness, fracturability, and resilience. Thus, the addition of polysaccharide hydrocolloids can effectively regulate the texture properties of meat products (especially the emulsified meat products), which mainly corresponds to improvements in gelling ability and water retention capacity of meat products. However, the excessive addition of polysaccharide hydrocolloids also causes the destabilizations of the microstructure, sensory quality, and texture of meat products especially upon heating, such as the fried beef patties [28].

Besides texture properties, the sensory receptivity is often evaluated in terms of color, odor, and taste. Zheng et al. [89] investigated the influence of amidated pectin as a lipid substitute on the quality of minced chicken breast (MCB). The MCB samples fortified with amidated pectin displayed higher hardness and L* values in comparison to the control group. After thermal processing, all MCB samples showed a close chroma, texture, electronic tongue sensory evaluation, and taste score, confirming the role of polysaccharide hydrocolloids as a fat substitute under the premise of ensuring the quality of meat products. Moreover, the application of films or coatings based on polysaccharide hydrocolloids can effectively maintain the sensory characteristics of meat products, especially for color preservation [83,90]. The myoglobin in meat products undergoes denaturation when exposed to ultraviolet radiation or oxygen, resulting in change of the color, while the implementation of protective layers can isolate the influence of oxygen and ultraviolet rays [100,101]. A chitosan-based coating was used to prolong the shelf life of turkey breast meat. This coating significantly improved the sensory properties of turkey breast meat, including increased scores of taste, odor, texture, and color. This enhancement was more pronounced with the extension of storage days [112]. Another example of such a protective layer is the tannic acid-grafted chitosan coating, which effectively retarded the oxidation of myoglobin, promoting the formation of high-iron myoglobin and preserving the stability of the red color of pork slices and simultaneously delaying odor production [113]. Additional coatings or films also exhibited comparable effects, such as gelatin-sodium alginate film incorporating beetroot peel extract [114] and carrageenan/alginate films [115].

### 3.5. Monitoring Freshness

**Figure 6:**
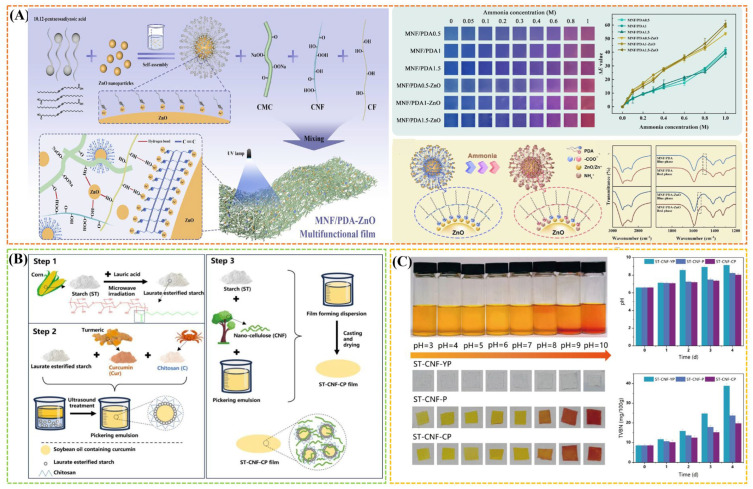
(**A**) Schematic diagram of MNF/PDA-ZnO film preparation as well as the color and ammonia response properties of films. Reproduced with permission from [117]; (**B**) schematic preparation diagram of the films by incorporating the chitosan-stabilized curcumin Pickering emulsion into the starch film matrix. Reproduced with permission from [116]; (**C**) colorimetric response of curcumin solution and films at different pH values as well as the results of monitoring pork freshness (pH and TVBN). Reproduced with permission from [116].

Recently, green intelligent food packaging using renewable and biodegradable biopolymers as materials and bioactive substances as functional materials has been widely studied. It possesses intelligent functions such as detection, sensing, recording, and tracking of food and its surroundings, thereby effectively monitoring food safety and food quality, as well as prolonging shelf life and providing early warnings about possible spoilage [116]. Polysaccharide hydrocolloids can be employed to fabricate intelligent packaging materials due to its film-forming, rich functional groups, and adsorption properties, which are capable of detecting the freshness of meat products [5]. Yue et al. [117] fabricated CMC-based multifunctional packaging film interspersed with wheat straw cellulose fibers (CF) and cellulose nanofibers (CNF) interacted by intermolecular hydrogen bonding. The films were then equipped with polydiacetylene (PDA)/ZnO nanoparticles (PDA/ZnO-NPs) to generate a highly sensitive specific response to biogenic amine as an ammonia colorimetric indicator film, which can provide effective quality retention and spoilage monitoring of high protein products, especially shrimp with high biogenic amine release. Moreover, the film also displayed high-strength, antimicrobial, UV-resistant, gas barrier, and water resistance properties (Figure 6A). In particular, the introduction of some bioactive substances to provide intelligent response properties is the main strategy. For example, a multifunctional active smart film was successfully fabricated by incorporating chitosan-adsorbed laurate esterified starch stabilized Pickering emulsion containing curcumin into the starch film matrix with nanocellulose as reinforcing agents (Figure 6B) [116]. The film showed good free radical scavenging activity and sustained high antioxidant capacity due to the presence of curcumin, and delayed pork spoilage with a slower rise in the pH and TVBN. Moreover, the curcumin also endowed the film with good pH-indicating ability to monitor the freshness of pork from the color of bright brown to saddle brown (Figure 6C) [116].

A recent study focused on the development of films using sweet potato starch (SPS), κ-carrageenan (KC), and *Oxalis triangularis* extract (OTE) that exhibited color changes in response to the increase of TVB-N content, thereby enabling the detection of beef freshness. Specifically, the color change was more obvious of SPS-KC-OTE-III film, which altered from purple to green as the TVB-N level increased on account of the highest anthocyanin content. SPS-KC-OTE films can be served as intelligent packaging films to monitor the freshness of meat products [118]. Additionally, a novel pH-responsive color indicator film was created by incorporating barberry anthocyanin (BA) into a composite film consisting of methylcellulose (MC) and chitosan nanofibers (ChNF), which had potential to apply as intelligent packaging material [34]. The color of the indicator film changed from reddish to pale peach when the protein of the lamb meat degraded after 72 h of storage, because volatile nitrogen compounds led to the change the pH level inside the package.

### 3.6. Providing Antibacterial Activity

Polysaccharide hydrocolloids have been widely studied and proved to have anti-foodborne pathogens activity, and their antibacterial effect is mainly achieved by destroying cell structure and impeding bioenergetics metabolism [119]. Compared with synthetic antimicrobials, polysaccharide hydrocolloids used as natural antibacterial agents conform to the increasingly popular food industry “cleaning label” and may also enhance the taste of meat products. It can be used as natural preservatives to replace synthetic additives in meat products, thus avoiding the adverse effects of using too many synthetic preservatives. Polysaccharide hydrocolloids are applied to meat products for anti-bacterial preservation currently mainly in the form of functional ingredients, antibacterial agents, or serving as food packaging (e.g., edible coating and film) [120]. The nanoemulsions prepared with chitosan showed a significant antibacterial effect on *E. coli* during the storage of minced beef and thus can be used as an antibacterial agent to extend the shelf life of meat products [121]. However, more studies have focused on the application of polysaccharide hydrocolloids in the field of antibacterial packaging of meat products. They can be grafted and synthesized into nanomaterials to augment the antimicrobial efficacy, which provides the basis for the preparation of composite films or coatings by polysaccharide hydrocolloids. For instance, Liu et al. [122] prepared a carboxymethyl chitosan/zinc alginate composite film (CMCS/SA-Zn) with a remarkable antimicrobial activity against *S. aureus* and *E. coli*, which can result in a prolonged shelf life of pork meat by 5 d. The zinc ions were uniformly distributed on the surface of the composite film through chelation to form zinc alginate and zinc hydroxide coatings. For both *E. coli* and *S. aureus*, the antibacterial effect of three groups of composite films CMCS/SA-Zn1, CMCS/SA-Zn2, and CMCS/SA-Zn3 was effective. Zinc alginate contact with bacteria caused a large part of the bacteria to be killed, and the release of zinc ions disrupted the growth and reproduction of bacteria. The total volatile basic nitrogen (TVB-N) content was also reduced, therefore reflecting the improved freshness of pork meat to some extent.

### 3.7. Ensuring Security

During thermal food processing such as smoking, deep-frying, and barbecuing, the formation of three primary carcinogens occurs: polycyclic aromatic hydrocarbons (PAHs), heterocyclic amines (HCA), and acrylamide (AA), which have harmful effects on the human respiratory system, nervous system, circulatory system, liver, and kidney [123]. The generation of these potentially carcinogenic compounds is more pronounced in deep-frying compared to alternative cooking techniques such as grilling or baking [57]. The natural cellulose aerogel-based adsorbent exhibited efficient adsorption capabilities for PAHs during smoked pork sausage manufacture that is installed over the smoke regulator, while maintained the desired quality characteristics of meat products at the same time. The adsorption efficacy for PAHs was affected by the surface structure and pore size of aerogels [123]. Moreover, Zhang et al. [124] studied the efficacy of polysaccharide hydrocolloids as inhibitors for HCA generation. The effects of six polysaccharide hydrocolloids, including alginic acid, sodium carboxymethyl cellulose (CMC-Na), chitosan, carrageenan, konjac glucomannan (KGM), and xanthan gum, on major HCA formation in roast beef patties as an ingredient were investigated. CMC-Na had the strongest inhibitory effect, followed by chitosan in roast beef patties. In addition, marination with CMC-Na solution had better inhibitory effects than powders on HCA formation. These findings suggest that polysaccharide hydrocolloids have the potential to impede the generation of mutagenic HCA in meat preparation and processing.

### 3.8. Improving Printability

**Figure 7:**
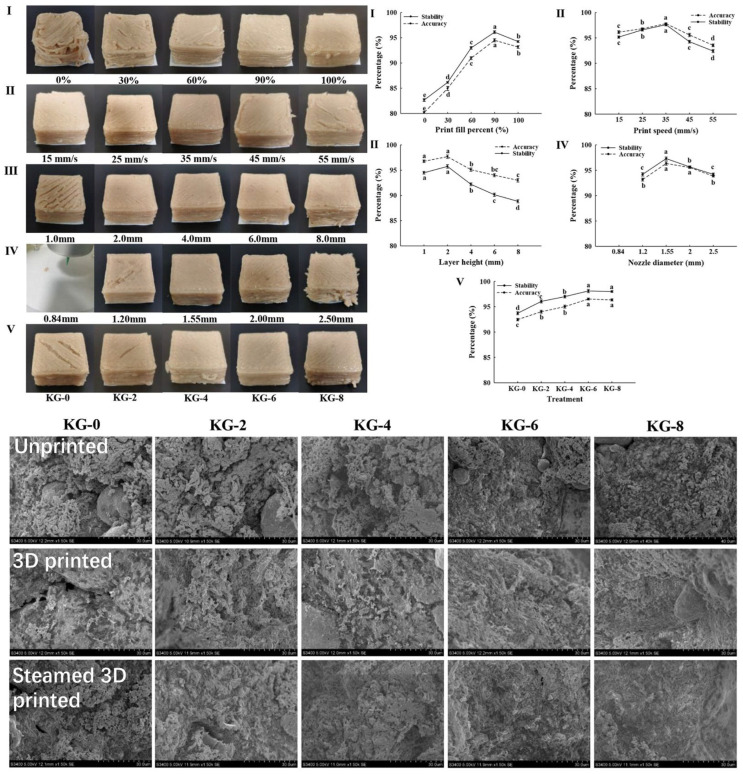
The pictures, accuracy and stability of 3D printed pork pastes. I, II, III, IV, and V present print fill percents, print speeds, layer heights, nozzle diameters, and KG addition levels (0–8 g/kg); micrographs of unprinted pork pastes, 3D-printed pork pastes, and steamed 3D-printed pork pastes. Reproduced with permission from [125].

Recently, 3D printing technology has become a research hotspot in the food industry because of its ability to develop personalized food, such as customized food for children, the elderly, pregnant women, soldiers, and patients with dysphagia [24]. Moreover, 3D printing has demonstrated its potential in minimizing raw material waste and mitigating the risk of food contamination. Extrusion, binder injection, and selective sintering 3D printing have emerged as the most prevalent techniques of 3D printing in the food industry [24]. In contrast to printable food materials, such as cream cheese and pizza dough, the direct printing of meat is infeasible, as it needs to be transformed into meat paste or batter before printing [24,66]. Generally, the common meat paste itself does not have suitable rheological properties to achieve 3D printing. The inclusion of polysaccharide hydrocolloids, such as carrageenan, xanthan gum, and sodium alginate, serving as thickeners or gelling agents, can effectively improve the 3D printability and self-supporting ability of meat pastes [125]. The improvement of rheological properties is also necessary to fulfill certain printability, thus the enhanced meat can be utilized as “ink” for printing purposes [25]. Xu et al. [126] confirmed the improvement of rheological properties and 3D printability of pork pastes by the addition of xanthan gum. The addition of 6 g/kg xanthan gum had excellent printing performance because the suitable incorporation of xanthan gum increased the water absorption ability, expansibility, and viscoelasticity. In the study of Dick, Bhandari, and Prakash [127], with the addition of 0.5% xanthan gum and 0.5% κ-carrageenan (sample XG/KG(1)), the cooked beef pastes possessed excellent printability and could be used as modified texture food, which was suitable for producing ready-to-eat meals and finger-foods for dysphagia diets colony with chewing and swallowing disparities due to the classification of IDDSI level 6 */7. Xu et al. [125] investigated effects of KG on the 3D printability of pork pastes. According to the results of visual analysis, accuracy, and stability, the best printing parameters are as follows: printing filling percent, 90%; printing speed, 35 mm/s; layer height, 2 mm; nozzle diameter, 1.55 mm, and KG addition level, 6 g/kg. The addition of KG can significantly improve the 3D printability and rheological properties of the pork pastes with a denser structure and smaller holes. Theoretically, KG can enhance a gel-like structure of meat protein through electrostatic interaction to improve the stability of the matrix (Figure 7).

Proteins as the sole material for 3D printing may still exhibit certain limitations, such as low solubility and susceptibility to pH/temperature variations. Usually, introducing polysaccharide can provide enhanced stability and improved properties compared to protein-based material alone [128]. Most polysaccharide hydrocolloids have an intrinsic nature to interact with protein, thus affecting protein properties such as stability, emulsification, solubility, gelling capacity, etc. It displays a great beneficial role in manufacturing meat analogs, including but not limited to promoting the formation of anisotropic structure, improving the texturization of protein fiber, water-holding capacity, and the color appearance, and influencing the flavor perception as well as acting as a fat analogue [129,130]. Dekkers, Nikiforidis, and Goot [131] found that filaments formed under the shear-induced action of pectin can be maintained in the continuous soybean isolate phase, and the addition of 4% pectin promoted the formation of fibrous anisotropic structures. Zhou et al. [132] used potato protein-based gels for the development of meat analogs, where pectin addition increased the gel malleability and formed a more porous and fibrous microstructure. For other polysaccharides, Leelapunnawut et al. [133] fabricated meat mimics through 3D printing from a mixture of pea protein isolate–alginate gel using κ-carrageenan as texture modifiers of 3D-printed meat mimics. Increasing κ-carrageenan content resulted in an increase of G′ and G″ of the printing material due to the formation of the polysaccharides–protein complex, and the texture properties of cooked meat mimics treated with 0.9% κ-carrageenan was closest to that of cooked salmon.

### 3.9. Enhancing Nutritional Properties

Fat in meat products provides abundant flavor and suitable texture, but excessive intake can influence human health and lead to obesity, chronic heart disease, arteriosclerosis, and so on. Hence, addressing the issue should reduce the elevated fat content in meat products for healthier alternatives, which can be achieved by introducing polysaccharide hydrocolloids [4,56,102]. Recently, the polysaccharide-based emulsion, emulsion gels, hydrogels, and oleogels and bigels have proved to be fat substitutes or fat mimetics that can simulate some of the desirable characteristics normally provided by fats in foods [29,56,134,135,136]. For examples, research has demonstrated the feasibility of substituting pork back fat in sausages with emulsion gel that formed by carrageenan and emulsion stabilized by zein/carboxymethyl dextrin, resulting in improved sausage hardness, viscoelasticity, and network structure [29]. A novel bigel-based fat substitute was fabricated using nanocellulose hydrogel and monoglyceride oleogel, and the replacement of butter with bigels in cookie preparation can efficiently reduce fat content without significantly altering the appearance or properties of the cookies [135]. Due to the strong interaction of polysaccharide hydrocolloids with other food components, the direct addition of polysaccharide can be used to fabricate fat replacers that can improve the food quality induced by fat [4]. Likewise, the addition of chitosan to the low-fat beef burgers as a fat substitute can improve the quality of the burger due to its high water-holding capacity and maintaining the color of the burger. And the lipid corruption has also been impeded. Moreover, with the increase of chitosan content, the hardness, elasticity, and viscosity of the burger were also significantly enhanced [137].

In addition to being fat alternatives, polysaccharide hydrocolloids also have the ability to reduce the amount of salt added to meat products. For instance, the combination of potassium chloride (KCl) and tapioca starch (TS) enables the reduction of salt levels to below 1.5% in sausages produced by high pressure processed pork (150 MPa) [138]. The inclusion of KCl in conjunction with TS to the sausage batters contributed to a favorable impact on the water-binding capability, which could be evidenced by a notable reduction in the total expressible fluid percentage and an increase in the water-holding capacity percentage. Furthermore, the sensory characteristics of the pork sausages remained largely unaffected by the introduction of KCl/TS. In another study, lipid and salt decreased fermented sausages, with fat content reduced by 25% and salt content reduced by 25% of KCl and 75% of NaCl, have been created by the utilization of the incorporation of microcrystalline cellulose, resistant starch, and oat fiber [139]. This not only provided consumers with healthier low fat and salt fermented sausages, but also exhibited no negative effect on the weight loss and sensory values and revealed antioxidant power as well as reinforced hardness and chewiness. Actually, the polysaccharide hydrocolloids also possess unique healthy characteristics in the form of dietary fiber and prebiotics [7].

**Table 2:** Beneficial roles of various polysaccharide hydrocolloids in meat products.

Types	Beneficial Roles in Meat Products	Advantages	References
Chitosan	Delaying lipid oxidation	The amount of malondialdehyde (MDA), 4-hydroxynonenal (4-HNE), and hexanal (HEX) was approximately 2-fold lower (*p* < 0.01) in the meat samples containing chitosan compared with control; chitosan reduced MDA formation by approximately 30% during in vitro digestion of beef patties.	[97]
Alginate films loaded CNC	Delaying lipid oxidation	The presence of CNC prevented the oxidation of lipids of chicken breast during the first 3 days of storage at 4 °C.	[17]
Chitosan-loaded nanoemulsion	Retarding protein oxidation	The carbonyl content in chitosan-loaded nanoemulsion treated turkey meat slices was less than 2 nmol/mg after 15 days of storage at 4 °C.	[103]
Sodium alginate- cellulose coating	Retarding protein oxidation	The total volatile base nitrogen (TVB-N) values of uncoated fresh pork were significantly higher than the sodium alginate-carboxymethyl cellulose-coated pork samples.	[140]
Nanocellulose	Acting as cryoprotectant	Nanocellulose significantly retarded the quick decrease of salt soluble protein content, Ca^2+^-ATPase activity and sulfhydryl content of myofibrillar protein in the frozen surimi.	[105]
Sodium alginate	Acting as Cryoprotectant	The ice production, thawing loss, and TVB-N value of samples with 1.0% sodium alginate ice glazing decreased by 28.30%, 21.02%, and 27.35%, while the chewiness and whiteness were increased by 15.02% and 10.40% after repeated freeze–thaw cycles.	[107]
Carrageenan	Improving sensory quality	Carrageenan significantly improved the textural and gel properties of frankfurters, as well as the rheological behaviour of meat batter.	[49]
Pectin	Improving sensory quality	Minced chicken breast samples fortified with amidated pectin showcased higher hardness and L* values in comparison to native pectin and the control group.	[89]
Chitosan- chitosan stabilized laurate esterified starch curcumin emulsion	Monitoring freshness	The curcumin endowed the film with pH indicating ability to indicate the freshness of pork from the color of bright brown to saddle brown.	[116]
Carboxymethyl cellulose based packaging film	Monitoring freshness	The films equipped with DA/ZnO generated a highly sensitive specific response to biogenic amine as an ammonia colorimetric indicator film.	[117]
Sodium alginate/chitosan-based intelligent bilayer film	Providing antibacterial activity	The bactericidal rates of the film towards *E. coli* and *S. aureus* were 99.23% and 97.49%, respectively. The film could extend the pork’s shelf life for 40 and 96 h at 20 °C and 4 °C.	[141]
Carboxymethyl chitosan/zinc alginate film	Providing antibacterial activity	The films displayed antimicrobial activity against *S. aureus* and *E. coli* and can prolong shelf life of pork meat by 5 d.	[122]
Carboxymethyl cellulose	Ensuring security	Marination with carboxymethyl cellulose in roast beef led to greater inhibitory effects on heterocyclic amines formation with the contents of PhIP, MeIQx and 4,8-DiMeIQx being reduced by over 90%, 80%, and 70%, respectively.	[124]
Carrageenan	Ensuring security	Carrageenan indicated significantly lower polar heterocyclic amines (including PhIP, MeIQx, MeIQ, IQ, 4,8-DiMeIQx, and 7,8-DiMeIQx) than the controls for the roasted tilapia fish patties.	[142]
Carrageenan	Improving printability	Carrageenan can significantly improve the 3D printability and rheological properties of the pork pastes with a denser structure and smaller holes.	[125]
Pectin	Improving printability	Pectin promoted the formation of fibrous anisotropic structures for meat analogues.	[131]
Nanocellulose	Enhancing nutritional properties	Nanocellulose hydrogel and monoglyceride oleogel based bigel as fat substitute of butter in cookie preparation can efficiently reduce fat content without significantly altering the appearance or properties of the cookies.	[135]
Starch	Enhancing nutritional properties	That use of the functional ingredient KCl/tapioca starch can reduce sodium levels in the manufacture of low-salt breakfast sausages to 1.0%.	[138]

## 4. Conclusions

This review provides a concise introduction to serval commonly used polysaccharide hydrocolloids (i.e., cellulose, chitosan, starch, sodium alginate, pectin, and carrageenan) in meat products and elucidates their properties and beneficia roles. Based on the recently published studies, the primary roles of polysaccharide hydrocolloids mainly include enhancing texture and color, retarding lipid and protein oxidation, extending shelf life, and regulating nutrient equilibrium. Additionally, polysaccharide hydrocolloids also exhibit a diverse array of prospective uses such as cryoprotection, mitigating the production of deleterious compounds, and facilitating the 3D printing of meat products.

Besides being served as outer packaging and monitoring materials, reformulation and functionalization of the meat products by adding polysaccharide hydrocolloids should holistically consider the nutritional, organoleptic, safety, and technological profile of the final products. Despite the extensive research conducted on the utilization of polysaccharide hydrocolloids in meat products with positive effects on improving the quality of meat products, there are still some critical issues for further exploration in the following areas: (i) developing polysaccharide hydrocolloids modification or multiple polysaccharide composites to enhance the covalent or non-covalent interactions with meat product proteins and clarifying the relevant interaction mechanism, so as to improve the ability of polysaccharide hydrocolloids to regulate texture, color protection, delay oxidation, and preservation of freshness; (ii) comprehending the mechanism of polysaccharide hydrocolloids functions as cryoprotectants and their potential applications in various meat products, including meat fillings and minced meat, to effectively impede quality deterioration; (iii) examining the viability of various polysaccharide hydrocolloids as binders for 3D printing of meat or meat analogue products by interacting with meat protein or plant protein, with the aim of enhancing the rheological and printing characteristics in order to create customized meat products; (iv) investigating the benefits of polysaccharide hydrocolloid based on materials in various applications such as carriers, fat substitutes, and packaging; (v) enhancing the feasibility and market acceptance of polysaccharide hydrocolloids as commercial fat substitutes, by applying advanced technology portfolios, achieving temperature-induced sol-gel transformation of fat substitutes, and clarifying their oxidation and digestion/metabolism properties. Finally, the mechanism of different polysaccharide hydrocolloids in various meat products should be systematically studied to preferably utilize polysaccharide hydrocolloids to facilitate the improvement of meat products quality. In summary, many challenging works still need to be carried out to excavate and exert the application potential of polysaccharide hydrocolloids in meat products.

## Figures and Tables

**Table 1:** Applications of polysaccharide hydrocolloids in different meat products.

Polysaccharide Hydrocolloids	Role of Polysaccharide Hydrocolloids	Research Highlights	Products	Applications	References
Carboxymethyl cellulose	Forming a film combined with zinc oxide nanoparticles and grape seed extract	Excellent antioxidant and antimicrobial ability, delaying lipid oxidation	High-fat meat products	Active food packaging	[22]
Ethyl cellulose	Forming an ethyl cellulose based oleogel added with adipic acid	Excellent oil binding capacity and satisfactory texture, color, and sensory properties	Beef burger	Acting as alternatives to hydrogenated vegetable shortening and animal fats	[32]
CNC	Replacing polyphosphates and starch	Affecting fat and water retention properties	Emulsion-type meat products	Functional food ingredient	[33]
Chitosan nanofiber	Forming a pH-responsive color indicator film loaded with barberry anthocyanins	Improving mechanical and water barrier abilities	Real-time monitoring of meat freshness	Freshness indicated packaging	[34]
Chitosan	Forming edible coatings	Controlling the growth of *S. aureus* and *L. monocytogenes*	Beef and mutton cuts	Enhancing meat safety	[35]
Chitosan	Replacing sodium nitrite	Improving water activity, color, sensory, and microbiological attributes	Fermented cooked sausages	Functional food ingredient	[36]
Chitosan	Preparing a eugenol/chitosan coating	Prolonging shelf life	Fresh pork loin meat	Edible fresh-keeping coating	[37]
Pea starches	Acting as an alternative to modified corn starch	Improving cook loss, water holding capacity, textural and eating quality	Low-fat bologna	Alternative ingredients for clean label products	[38]
Modified cassava starch	Forming a starch film incorporated with papain	Lowering water sensitivity and solubility, and raising the tenderness	Beef	Active meat packaging	[39]
Corn starch	Forming a starch film incorporated with red cabbage extract	Exhibiting antioxidant capacity and delaying quality deterioration	Ground meat	Active meat packaging	[40]
Modified rice starch	Forming a biodegradable film loaded with curcumin	Detecting the generation of hypoxanthine	Chicken	Active meat packaging	[41]
Acetylated corn starch	As fat substitute for making beef patties	Improving the physicochemical quality and the sensory	Beef patties	Fat replacers in meat products	[42]
Sodium alginate	Replacing pork back-fat	Adjusting physical-chemical, protein conformation and sensory	Low-fat frankfurters	Fat replacers in meat products	[33]
Sodium alginate	Forming agar-sodium alginate (AS) bilayer antibacterial film incorporated with ginger essential oil	Delaying lipid oxidation and protein decomposition and inhibiting microbial contamination	Beef refrigeration	Antibacterial active packaging material	[43]
Sodium alginate	Preparing an edible film loaded with pineapple peel extract	Inhibiting microbial growth and maintaining the color and retarding lipid oxidation	Beef	Active packaging film for meat preservation	[44]
Pectin	Forming structurally different pectin combined with polyphenol	Inhibiting protein oxidation and heterocyclic amines	Meat-based snacks	Functional food ingredient	[45]
Pectin	Preparing the low-fat Chinese sausage as a novel fat replacer	Enhancing color and conserving the physical qualities as well as sensory attributes	Dried Chinese sausage	Fat replacers in meat products	[46]
Pectin	Forming a coating with nisin	Inhibiting *L. monocytogenes* of turkey bologna	Deli meat turkey bologna	Antibacterial packaging coating	[47]
κ-carrageenan	Improving the quality of meat products	Improving the stability and gel properties of meat batters before and after heat treatment	Meat batter	Functional food ingredient	[48]
κ-carrageenan	Improving the quality of emulsified meat products	Enhancing the texture, and rheological characteristic, and reducing in vitro digestibility of frankfurters	Frankfurters	As quality improvers for frankfurters	[49]

## Data Availability

No new data were created or analyzed in this study. Data sharing is not applicable to this article.

## References

[1] Wijaya W., Patel A.R., Setiowati A.D., Van der Meeren P. (2017). Functional colloids from proteins and polysaccharides for food applications. Trends Food Sci. Technol..

[2] Li Z., Lin Z. (2021). Recent advances in polysaccharide-based hydrogels for synthesis and applications. Aggregate.

[3] Cui F., Zhao S., Guan X., McClements D.J., Liu X., Liu F., Ngai T. (2021). Polysaccharide-based Pickering emulsions: Formation, stabilization and applications. Food Hydrocoll..

[4] Wang J., Shang M., Li X., Sang S., McClements D.J., Chen L., Long J., Jiao A., Ji H., Jin Z. (2023). Polysaccharide-based colloids as fat replacers in reduced-fat foods. Trends Food Sci. Technol..

[5] Wang Y., Liu K., Zhang M., Xu T., Du H., Pang B., Si C. (2023). Sustainable polysaccharide-based materials for intelligent packaging. Carbohydr. Polym..

[6] Fernando S.S., Jo C., Mudannayake D.C., Jayasena D.D. (2024). An overview of the potential application of chitosan in meat and meat products. Carbohydr. Polym..

[7] Kaur R., Sharma M. (2019). Cereal polysaccharides as sources of functional ingredient for reformulation of meat products: A review. J. Funct. Foods.

[8] Marchetti L., Andrés S.C. (2021). Use of nanocellulose in meat products. Curr. Opin. Food Sci..

[9] Udo T., Mummaleti G., Mohan A., Singh R.K., Kong F. (2023). Current and emerging applications of carrageenan in the food industry. Food Res. Int..

[10] Yang X., Li A., Li X., Sun L., Guo Y. (2020). An overview of classifications, properties of food polysaccharides and their links to applications in improving food textures. Trends Food Sci. Technol..

[11] Zhou L., Zhang W., Wang J. (2022). Recent advances in the study of modified cellulose in meat products: Modification method of cellulose, meat quality improvement and safety concern. Trends Food Sci. Technol..

[12] Zhao N., Zou H., Sun S., Yu C. (2020). The interaction between sodium alginate and myofibrillar proteins: The rheological and emulsifying properties of their mixture. Int. J. Biol. Macromol..

[13] Cao C., Liang X., Xu Y., Kong B., Sun F., Liu H., Zhang H., Liu Q., Wang H. (2024). Effects and mechanisms of different κ-carrageenan incorporation forms and ionic strength on the physicochemical and gelling properties of myofibrillar protein. Int. J. Biol. Macromol..

[14] Thangavelu K.P., Kerry J.P., Tiwari B.K., McDonnell C.K. (2019). Novel processing technologies and ingredient strategies for the reduction of phosphate additives in processed meat. Trends Food Sci. Technol..

[15] Fraqueza M.J., Laranjo M., Elias M., Patarata L. (2021). Microbiological hazards associated with salt and nitrite reduction in cured meat products: Control strategies based on antimicrobial effect of natural ingredients and protective microbiota. Curr. Opin. Food Sci..

[16] Gheorghita Puscaselu R., Gutt G., Amariei S. (2020). The use of edible films based on sodium alginate in meat product packaging: An eco-friendly alternative to conventional plastic materials. Coatings.

[17] Criado P., Fraschini C., Salmieri S., Lacroix M. (2020). Cellulose nanocrystals (CNCs) loaded alginate films against lipid oxidation of chicken breast. Food Res. Int..

[18] Xiao L., Kang S., Lapu M., Jiang P., Wang X., Liu D., Li J., Liu M. (2022). Preparation and characterization of chitosan/pullulan film loading carvacrol for targeted antibacterial packaging of chilled meat. Int. J. Biol. Macromol..

[19] Kumar S., Mukherjee A., Dutta J. (2020). Chitosan based nanocomposite films and coatings: Emerging antimicrobial food packaging alternatives. Trends Food Sci. Technol..

[20] Swarupa S., Thareja P. (2024). Techniques, applications and prospects of polysaccharide and protein based biopolymer coatings: A review. Int. J. Biol. Macromol..

[21] Fang Z., Lin D., Warner R.D., Ha M. (2018). Effect of gallic acid/chitosan coating on fresh pork quality in modified atmosphere packaging. Food Chem..

[22] Priyadarshi R., Kim S., Rhim J. (2021). Carboxymethyl cellulose-based multifunctional film combined with zinc oxide nanoparticles and grape seed extract for the preservation of high-fat meat products. Sustain. Mater. Technol..

[23] Sungsinchai S., Niamnuy C., Wattanapan P., Charoenchaitrakool M., Devahastin S. (2019). Texture modification technologies and their opportunities for the production of dysphagia foods: A review. Compr. Rev. Food Sci. Food Saf..

[24] Dong H., Wang P., Yang Z., Xu X. (2023). 3D printing based on meat materials: Challenges and opportunities. Curr. Res. Food Sci..

[25] Yu X., Wang Y., Zhao W., Li S., Pan J., Prakash S., Dong X. (2023). Hydrophilic colloids (Konjac gum/Xanthan gum) in 3D printing of transitional food from fish paste. Food Hydrocoll..

[26] Chattopadhyay K., Xavier K.A.M., Layana P., Balange A.K., Nayak B.B. (2019). Chitosan hydrogel inclusion in fish mince based emulsion sausages: Effect of gel interaction on functional and physicochemical qualities. Int. J. Biol. Macromol..

[27] Zhao X., Guo R., Li X., Wang X., Zeng L., Wen X., Huang Q. (2023). Effect of oil-modified crosslinked starch as a new fat replacer on gel properties, water distribution, and microstructures of pork meat batter. Food Chem..

[28] Gibis M., Schuh V., Weiss J. (2015). Effects of carboxymethyl cellulose (CMC) and microcrystalline cellulose (MCC) as fat replacers on the microstructure and sensory characteristics of fried beef patties. Food Hydrocoll..

[29] Li X., Meng R., Xu B., Zhang B., Cui B., Wu Z. (2022). Function emulsion gels prepared with carrageenan and zein/carboxymethyl dextrin stabilized emulsion as a new fat replacer in sausages. Food Chem..

[30] Gao X., Pourramezan H., Ramezan Y., Roy S., Zhang W., Assadpour E., Zou J., Jafari S.M. (2024). Application of gums as techno-functional hydrocolloids in meat processing and preservation: A review. Int. J. Biol. Macromol..

[31] Sun X., Wu Y., Song Z., Chen X. (2022). A review of natural polysaccharides for food cryoprotection: Ice crystals inhibition and cryo-stabilization. Bioact. Carbohydr. Diet. Fibre.

[32] Adili L., Roufegarinejad L., Tabibiazar M., Hamishehkar H., Alizadeh A. (2020). Development and characterization of reinforced ethyl cellulose based oleogel with adipic acid: Its application in cake and beef burger. LWT.

[33] Parés D., Pèlach M.À., Toldrà M., Saguer E., Tarrés Q., Carretero C. (2018). Nanofibrillated cellulose as functional ingredient in emulsion-type meat products. Food Bioprocess Technol..

[34] Alizadeh-Sani M., Tavassoli M., Mohammadian E., Ehsani A., Khaniki G.J., Priyadarshi R., Rhim J. (2021). pH-responsive color indicator films based on methylcellulose/chitosan nanofiber and barberry anthocyanins for real-time monitoring of meat freshness. Int. J. Biol. Macromol..

[35] Economou V., Tsitsos A., Theodoridis A., Ambrosiadis I., Arsenos G. (2022). Effects of chitosan coatings on controlling listeria monocytogenes and methicillin-resistant *Staphylococcus aureus* in beef and mutton cuts. Appl. Sci..

[36] Ozaki M.M., Munekata P.E.S., Lopes A.D.S., Nascimento M.D.S.D., Pateiro M., Lorenzo J.M., Pollonio M.A.R. (2020). Using chitosan and radish powder to improve stability of fermented cooked sausages. Meat Sci..

[37] Zhao R., Zhang Y., Chen H., Song R., Li Y. (2022). Performance of eugenol emulsion/chitosan edible coating and application in fresh meat preservation. J. Food Process Preserv..

[38] Pietrasik Z., Soladoye O.P. (2021). Use of native pea starches as an alternative to modified corn starch in low-fat bologna. Meat Sci..

[39] Wongphan P., Khowthong M., Supatrawiporn T., Harnkarnsujarit N. (2022). Novel edible starch films incorporating papain for meat tenderization. Food Packag. Shelf Life.

[40] Ribeiro Sanches M.A., Camelo-Silva C., Da Silva Carvalho C., Rafael De Mello J., Barroso N.G., Lopes Da Silva Barros E., Silva P.P., Pertuzatti P.B. (2021). Active packaging with starch, red cabbage extract and sweet whey: Characterization and application in meat. LWT.

[41] Erna K.H., Felicia W.X.L., Rovina K., Vonnie J.M., Huda N. (2022). Development of curcumin/rice starch films for sensitive detection of hypoxanthine in chicken and fish meat. Carbohydr. Polym. Technol. Appl..

[42] Eshag Osman M.F., Mohamed A.A., Mohamed Ahmed I.A., Alamri M.S., Al Juhaimi F.Y., Hussain S., Ibraheem M.A., Qasem A.A. (2022). Acetylated corn starch as a fat replacer: Effect on physiochemical, textural, and sensory attributes of beef patties during frozen storage. Food Chem..

[43] Zhang B., Liu Y., Peng H., Lin Y., Cai K. (2023). Effects of ginger essential oil on physicochemical and structural properties of agar-sodium alginate bilayer film and its application to beef refrigeration. Meat Sci..

[44] Lourenço S.C., Fraqueza M.J., Fernandes M.H., Moldão-Martins M., Alves V.D. (2020). Application of edible alginate films with pineapple peel active compounds on beef meat preservation. Antioxidants.

[45] Snel S.J.E., Otto K., Schlangen M., Beyrer M., van der Goot A.J. (2024). Type of pectin determines structuring potential of soy proteins into meat analogue applications. Food Hydrocoll..

[46] Wongkaew M., Sommano S.R., Tangpao T., Rachtanapun P., Jantanasakulwong K. (2020). Mango peel pectin by microwave-assisted extraction and its use as fat replacement in dried Chinese sausage. Foods.

[47] Morgan A., Darby D., Bruce T., Romero A., Cooksey K. (2022). Development of an antimicrobial coating containing nisin and pectin for deli meat turkey bologna. LWT.

[48] Feng Y., Liang X., Zhang J., Kong B., Shi P., Cao C., Zhang H., Liu Q., Zhang Y. (2024). Effects of transglutaminase coupled with κ-carrageenan on the rheological behaviours, gel properties and microstructures of meat batters. Food Hydrocoll..

[49] Cao C., Yuan D., Kong B., Chen Q., He J., Liu Q. (2022). Effect of different κ-carrageenan incorporation forms on the gel properties and in vitro digestibility of frankfurters. Food Hydrocoll..

[50] Aziz T., Farid A., Haq F., Kiran M., Ullah A., Zhang K., Li C., Ghazanfar S., Sun H., Ullah R. (2022). A Review on the Modification of Cellulose and Its Applications. Polymers.

[51] Tian H., Li W., Chen C., Yu H., Yuan H. (2023). Antibacterial absorbent mat based on oxidized bacterial nanocellulose for chilled meat preservation. Food Packag. Shelf Life.

[52] Yuan H., Li W., Chen C., Yu H., Huang J., Lou X., Tian H. (2023). The role of bacterial nanocellulose mats encapsulated with cinnamaldehyde on chilled meat preservation. Int. J. Food Sci. Technol..

[53] Bohrer B.M., Izadifar M., Barbut S. (2023). Structural and functional properties of modified cellulose ingredients and their application in reduced-fat meat batters. Meat Sci..

[54] Zhang H., Tian X., Zhang K., Du Y., Guo C., Liu X., Wang Y., Xing J., Wang W. (2022). Influence of content and degree of substitution of carboxymethylated cellulose nanofibrils on the gelation properties of cull cow meat myofibrillar proteins. LWT.

[55] Song Z., Ma T., Zhi X., Du B. (2021). Cellulosic films reinforced by chitosan-citric complex for meat preservation: Influence of nonenzymatic browning. Carbohydr. Polym..

[56] Wang Y., Wang W., Jia H., Gao G., Wang X., Zhang X., Wang Y. (2018). Using Cellulose Nanofibers and Its Palm Oil Pickering Emulsion as Fat Substitutes in Emulsified Sausage. J. Food Sci..

[57] Wang Z., Ng K., Warner R.D., Stockmann R., Fang Z. (2023). Application of cellulose- and chitosan-based edible coatings for quality and safety of deep-fried foods. Compr. Rev. Food Sci. Food Saf..

[58] Zhang R., Ma S., Li L., Zhang M., Tian S., Wang D., Liu K., Liu H., Zhu W., Wang X. (2021). Comprehensive utilization of corn starch processing by-products: A review. Grain Oil Sci. Technol..

[59] Rahmadi Putri T., Adhitasari A., Paramita V., Endy Yulianto M., Dwi Ariyanto H. (2023). Effect of different starch on the characteristics of edible film as functional packaging in fresh meat or meat products: A review. Mater. Today Proc..

[60] Goksen G., Demir D., Echegaray N., Bangar S.P., Gomes Da Cruz A., Shao P., Lin Y., Lorenzo J.M. (2024). New insights of active and smart natural-based electrospun mats for food safety in meat and meat products. Food Biosci..

[61] Gagaoua M., Pinto V.Z., Göksen G., Alessandroni L., Lamri M., Dib A.L., Boukid F. (2022). Electrospinning as a promising process to preserve the quality and safety of meat and meat products. Coatings.

[62] Kong L., Ziegler G.R. (2014). Fabrication of pure starch fibers by electrospinning. Food Hydrocoll..

[63] Cui H., Lu J., Li C., Lin L. (2021). Fabrication of phospholipid nanofibers containing eugenol@cationic starch nanoparticles against Bacillus cereus in beef. LWT.

[64] Hernández-García E., Vargas M., Chiralt A. (2022). Starch-polyester bilayer films with phenolic acids for pork meat preservation. Food Chem..

[65] Zhao Y., Teixeira J.S., Saldaña M.D.A., Gänzle M.G. (2019). Antimicrobial activity of bioactive starch packaging films against Listeria monocytogenes and reconstituted meat microbiota on ham. Int. J. Food Microbiol..

[66] Liu P., Dang X., Woo M.W., Chattha S.A., An J., Shan Z. (2022). Feasibility study of starch-based biomass incorporated 3d printed beef. Starch-Stärke.

[67] Li H., Qu S., Ma P., Zhang J., Zhao K., Chen L., Huang Q., Zou G., Tang H. (2023). Effects of chitosan coating combined with thermal treatment on physicochemical properties, bacterial diversity and volatile flavor of braised duck meat during refrigerated storage. Food Res. Int..

[68] Afroz Ali S.M., Niaz T., Munir A., Shahid R., Shabbir S., Noor T., Imran M. (2023). Potential of pectin-chitosan based composite films embedded with quercetin-loaded nanofillers to control meat associated spoilage bacteria. Food Biosci..

[69] Ways T.M.M., Lau W.M., Khutoryanskiy V.V. (2018). Chitosan and its derivatives for application in mucoadhesive drug delivery systems. Polymers.

[70] Arkoun M., Daigle F., Heuzey M., Ajji A. (2017). Mechanism of action of electrospun chitosan-based nanofibers against meat spoilage and pathogenic bacteria. Molecules.

[71] Tamzid F., Sakhawat S.B., Rashid T.U. (2024). Chitosan based electrospun nanofibrous materials: A sustainable alternative for food packaging. Trends Food Sci. Technol..

[72] Cui H., Bai M., Li C., Liu R., Lin L. (2018). Fabrication of chitosan nanofibers containing tea tree oil liposomes against *Salmonella* spp. in chicken. LWT.

[73] Zheng K., Li B., Liu Y., Wu D., Bai Y., Xiang Q. (2023). Effect of chitosan coating incorporated with oregano essential oil on microbial inactivation and quality properties of refrigerated chicken breasts. LWT.

[74] Duran A., Kahve H.I. (2020). The effect of chitosan coating and vacuum packaging on the microbiological and chemical properties of beef. Meat Sci..

[75] Zhang M., Luo W., Yang K., Li C. (2022). Effects of sodium alginate edible coating with cinnamon essential oil nanocapsules and nisin on quality and shelf life of beef slices during refrigeration. J. Food Prot..

[76] Alirezalu K., Moazami Goodarzi A.H., Roufegarinejad L., Yaghoubi M., Lorenzo J.M. (2022). Combined effects of calcium-alginate coating and Artemisia fragrance essential oil on chicken breast meat quality. Food Sci. Nutr..

[77] Hanula M., Szpicer A., Górska-Horczyczak E., Khachatryan G., Pogorzelski G., Pogorzelska-Nowicka E., Poltorak A. (2022). Hydrogel emulsion with encapsulated safflower oil enriched with açai extract as a novel fat substitute in beef burgers subjected to storage in cold conditions. Molecules.

[78] Kang Z., Wang T., Li Y., Li K., Ma H. (2020). Effect of sodium alginate on physical-chemical, protein conformation and sensory of low-fat frankfurters. Meat Sci..

[79] Kim T., Yong H., Jung S., Kim Y., Choi Y. (2020). Effects of replacing pork fat with grape seed oil and gelatine/alginate for meat emulsions. Meat Sci..

[80] Atilgan E., Kilic B. (2017). Effects of microbial transglutaminase, fibrimex and alginate on physicochemical properties of cooked ground meat with reduced salt level. J. Food Sci. Technol..

[81] Shi H., Zhang X., Chen X., Fang R., Zou Y., Wang D., Xu W. (2020). How ultrasound combined with potassium alginate marination tenderizes old chicken breast meat: Possible mechanisms from tissue to protein. Food Chem..

[82] Choi I., Lee Y., Lyu J.S., Lee J., Han J. (2022). Characterization of ionically crosslinked alginate films: Effect of different anion-based metal cations on the improvement of water-resistant properties. Food Hydrocoll..

[83] Alexandre S., Vital A.C.P., Mottin C., Do Prado R.M., Ornaghi M.G., Ramos T.R., Guerrero A., Pilau E.J., Do Prado I.N. (2021). Use of alginate edible coating and basil (*Ocimum* spp.) extracts on beef characteristics during storage. J. Food Sci. Technol..

[84] Nastasi J.R., Kontogiorgos V., Daygon V.D., Fitzgerald M.A. (2022). Pectin-based films and coatings with plant extracts as natural preservatives: A systematic review. Trends Food Sci. Technol..

[85] Kumar S., Reddy A.R.L., Basumatary I.B., Nayak A., Dutta D., Konwar J., Purkayastha M.D., Mukherjee A. (2023). Recent progress in pectin extraction and their applications in developing films and coatings for sustainable food packaging: A review. Int. J. Biol. Macromol..

[86] Moll P., Salminen H., Schmitt C., Weiss J. (2023). Pea protein–sugar beet pectin binders can provide cohesiveness in burger type meat analogues. Eur. Food Res. Technol..

[87] Byun C., Zheng Y., Pierce A., Wagner W.L., Scheller H.V., Mohnen D., Ackermann M., Mentzer S.J., Lawrence Berkeley National Lab LBNL B.C.U.S. (2019). The effect of calcium on the cohesive strength and flexural properties of low-methoxyl pectin biopolymers. Molecules.

[88] Moll P., Salminen H., Stadtmueller L., Schmitt C., Weiss J. (2022). Comparison of binding properties of a laccase-treated pea protein-sugar beet pectin mixture with methylcellulose in a bacon-type meat analogue. Foods.

[89] Zheng C., Cai N., Huang C., Huang Y., Zou J., Zhang G., Fei P. (2023). Evaluation of amidated pectin as fat substitutes for minced chicken breast: Physicochemical properties and edible quality. Food Res. Int..

[90] Sani I.K., Geshlaghi S.P., Pirsa S., Asdagh A. (2021). Composite film based on potato starch/apple peel pectin/ZrO_2_ nanoparticles/microencapsulated Zataria multiflora essential oil; investigation of physicochemical properties and use in quail meat packaging. Food Hydrocoll..

[91] Shin D., Yune J.H., Kim Y.J., Keum S.H., Jung H.S., Kwon H.C., Kim D.H., Sohn H., Jeong C.H., Lee H.G. (2022). Effects of duck fat and κ-carrageenan as replacements for beef fat and pork backfat in frankfurters. Anim. Biosci..

[92] Domínguez R., Pateiro M., Gagaoua M., Barba F.J., Zhang W., Lorenzo J.M. (2019). A comprehensive review on lipid oxidation in meat and meat products. Antioxidants.

[93] Huang X., Ahn D.U. (2019). Lipid oxidation and its implications to meat quality and human health. Food Sci. Biotechnol..

[94] Amaral A.B., Silva M.V.D., Lannes S.C.D.S. (2018). Lipid oxidation in meat: Mechanisms and protective factors—A review. Food Sci. Technol..

[95] Chaudhary S., Kumar V., Sharma V., Sharma R., Kumar S. (2022). Chitosan nanoemulsion: Gleam into the futuristic approach for preserving the quality of muscle foods. Int. J. Biol. Macromol..

[96] Liu J., Chen B., Hu Q., Zhang Q., Huang B., Fei P. (2023). Pectin grafted with resorcinol and 4-hexylresorcinol: Preparation, characterization and application in meat preservation. Int. J. Biol. Macromol..

[97] Han M., Clausen M.P., Christensen M., Vossen E., Van Hecke T., Bertram H.C. (2018). Enhancing the health potential of processed meat: The effect of chitosan or carboxymethyl cellulose enrichment on inherent microstructure, water mobility and oxidation in a meat-based food matrix. Food Funct..

[98] El Sheikha A.F., Allam A.Y., ElObeid T., Basiouny E.A., Abdelaal A.A., Amarowicz R., Oz E., Proestos C., Karrar E., Oz F. (2022). Impact of a carboxymethyl cellulose coating incorporated with an ethanolic propolis extract on the quality criteria of chicken breast meat. Antioxidants.

[99] Zhang L., Li Q., Bao Y., Tan Y., Lametsch R., Hong H., Luo Y. (2024). Recent advances on characterization of protein oxidation in aquatic products: A comprehensive review. Crit. Rev. Food Sci. Nutr..

[100] Nawaz A., Irshad S., Ali Khan I., Khalifa I., Walayat N., Muhammad Aadil R., Kumar M., Wang M., Chen F., Cheng K. (2022). Protein oxidation in muscle-based products: Effects on physicochemical properties, quality concerns, and challenges to food industry. Food Res. Int..

[101] Song J., Jiang L., Peng H., Qi M., Zhang M., Qi J., Ma C., Li H., Zhang D. (2022). Microcapsule prepared by extruding starch and procyanidins inhibited protein oxidation and improved quality of chicken sausages. LWT.

[102] Mousa R.M.A. (2021). Development of 95% fat-free hamburgers using binary and ternary composites from polysaccharide hydrocolloids and fruit peel flours as fat replacer systems. J. Food Process Preserv..

[103] Keykhosravy K., Khanzadi S., Hashemi M., Azizzadeh M. (2022). Protective effect of chitosan-loaded nanoemulsion containing Zataria multiflora Boiss and Bunium persicum Boiss essential oils as coating on lipid and protein oxidation in chill stored turkey breast fillets. J. Food Sci..

[104] Li T., Li M., Zhong Q., Wu T. (2020). Effect of fibril length on the ice recrystallization inhibition activity of nanocelluloses. Carbohydr. Polym..

[105] Li Z., Wang Q., Li S., Chang Y., Zheng X., Cao H., Zheng Y. (2022). Usage of nanocrystalline cellulose as a novel cryoprotective substance for the *Nemipterus virgatus* surimi during frozen storage. Food Chem. X.

[106] Tan M., Ding Z., Yang D., Xie J. (2022). The quality properties of frozen large yellow croaker fillets during temperature fluctuation cycles: Improvement by cellobiose and carboxylated cellulose nanofibers. Int. J. Biol. Macromol..

[107] Li W., Bai X., Xia X., Chen H. (2024). Effect of sodium alginate ice glazing on the quality of the freeze-thawed fish balls. Int. J. Biol. Macromol..

[108] Coria-Hernández J., Méndez-Albores A., Arjona-Román J.L., Meléndez-Pérez R. (2022). Ultrasound-assisted diffusion of waxy starch cryogel on frozen-stored pork meat. LWT.

[109] Zhang H., Li X., Sun S., Wang Y., Li Z., Kang H., Peng X. (2023). Effects of carboxymethyl chitosan on the oxidation stability and gel properties of myofibrillar protein from frozen pork patties. Int. J. Biol. Macromol..

[110] Pematilleke N., Kaur M., Adhikari B., Torley P.J. (2022). Investigation of the effects of addition of carboxy methyl cellulose (CMC) and tapioca starch (TS) on the beef patties targeted to the needs of people with dysphagia: A mixture design approach. Meat Sci..

[111] Piao X., Li J., Zhao Y., Guo L., Zheng B., Zhou R., Ostrikov K.K. (2022). Oxidized cellulose nanofibrils-based surimi gel enhancing additives: Interactions, performance and mechanisms. Food Hydrocoll..

[112] Sayadi M., Langroodi A.M., Pourmohammadi K. (2021). Combined effects of chitosan coating incorporated with Berberis vulgaris extract and Mentha pulegium essential oil and MAP in the shelf life of turkey meat. J. Food Meas. Charact..

[113] Zhang H., Li X., Kang H., Peng X. (2022). Effect of tannic acid-grafted chitosan coating on the quality of fresh pork slices during cold storage. Meat Sci..

[114] Chaari M., Elhadef K., Akermi S., Ben Akacha B., Fourati M., Chakchouk Mtibaa A., Ennouri M., Sarkar T., Shariati M.A., Rebezov M. (2022). Novel active food packaging films based on gelatin-sodium alginate containing beetroot peel extract. Antioxidants.

[115] Khan A., Priyadarshi R., Bhattacharya T., Rhim J. (2023). Carrageenan/alginate-based functional films incorporated with allium sativum carbon dots for UV-barrier food packaging. Food Bioprocess Technol..

[116] Yang Z., Chen Q., Wei L. (2024). Active and smart biomass film with curcumin Pickering emulsion stabilized by chitosan-adsorbed laurate esterified starch for meat freshness monitoring. Int. J. Biol. Macromol..

[117] Yue C., Wang M., Zhou Z., You Y., Wang G., Wu D. (2024). Cellulose-based intelligent packaging films with antibacterial, UV-blocking, and biodegradable properties for shrimp freshness monitoring. Chem. Eng. J..

[118] Jiang C., Liu T., Wang S., Zou Y., Cao J., Wang C., Hang C., Jin L. (2023). Antioxidant and ammonia-sensitive films based on starch, κ-carrageenan and Oxalis triangularis extract as visual indicator of beef meat spoilage. Int. J. Biol. Macromol..

[119] Wang Z., Sun Q., Zhang H., Wang J., Fu Q., Qiao H., Wang Q. (2021). Insight into antibacterial mechanism of polysaccharides: A review. LWT.

[120] Ben Akacha B., Michalak M., Najar B., Venturi F., Taglieri I., Kačániová M., Ben Saad R., Mnif W., Garzoli S., Ben Hsouna A. (2023). Recent advances in the incorporation of polysaccharides with antioxidant and antibacterial functions to preserve the quality and shelf life of meat products. Foods.

[121] Lotfy T.M.R., Shawir S.M.S., Badawy M.E.I. (2023). The impacts of chitosan-essential oil nanoemulsions on the microbial diversity and chemical composition of refrigerated minced meat. Int. J. Biol. Macromol..

[122] Liu W., Kang S., Xue J., Chen S., Yang W., Yan B., Liu D. (2023). Self-assembled carboxymethyl chitosan/zinc alginate composite film with excellent water resistant and antimicrobial properties for chilled meat preservation. Int. J. Biol. Macromol..

[123] Kim D., Han G., Shin H. (2021). Adsorption of polycyclic aromatic hydrocarbons (PAHs) by cellulosic aerogels during smoked pork sausage manufacture. Food Control.

[124] Zhang N., Zhao Y., Fan D., Xiao J., Cheng K., Wang M. (2020). Inhibitory effects of some hydrocolloids on the formation of heterocyclic amines in roast beef. Food Hydrocoll..

[125] Xu J., Fan Y., Chen Q., Sun F., Li M., Kong B., Xia X. (2023). Effects of κ-carrageenan gum on 3D printability and rheological properties of pork pastes. Meat Sci..

[126] Xu J., Fan Y., Liu H., Liu Q., Zhamsaranova S., Kong B., Chen Q. (2023). Improvement of rheological properties and 3D printability of pork pastes by the addition of xanthan gum. LWT.

[127] Dick A., Bhandari B., Prakash S. (2021). Printability and textural assessment of modified-texture cooked beef pastes for dysphagia patients. Future Foods.

[128] Liu Y., Zhang Y., Cai L., Zeng Q., Wang P. (2024). Protein and protein-polysaccharide composites-based 3D printing: The properties, roles and opportunities in future functional foods. Int. J. Biol. Macromol..

[129] Zhang J., Liu P., Wu A., Song Y., Li Q., Liao X., Zhao J. (2024). Towards understanding pectin-protein interaction and the role of pectin in plant-based meat analogs constructing. LWT.

[130] Huang M., Mehany T., Xie W., Liu X., Guo S., Peng X. (2022). Use of food carbohydrates towards the innovation of plant-based meat analogs. Trends Food Sci. Technol..

[131] Dekkers B.L., Nikiforidis C.V., van der Goot A.J. (2016). Shear-induced fibrous structure formation from a pectin/SPI blend. Innov. Food Sci. Emerg. Technol..

[132] Zhou H., Hu X., Xiang X., McClements D.J. (2023). Modification of textural attributes of potato protein gels using salts, polysaccharides, and transglutaminase: Development of plant-based foods. Food Hydrocoll..

[133] Leelapunnawut S., Ngamwonglumlert L., Devahastin S., Derossi A., Caporizzi R., Chiewchan N. (2022). Effects of texture modifiers on physicochemical properties of 3d-printed meat mimics from pea protein isolate-alginate gel mixture. Foods.

[134] Lin Q., Jiang L., Li X., Sang S., Ji H., Jin Z., Qiu C. (2024). Starch based fat replacers in food system: Modification, structured design, and application. Food Biosci..

[135] Hou Y., Wu Y., Ouyang J. (2024). Novel bigel based on nanocellulose hydrogel and monoglyceride oleogel: Preparation, characteristics and application as fat substitute. Food Res. Int..

[136] Ghiasi F., Golmakani M. (2022). Fabrication and characterization of a novel biphasic system based on starch and ethylcellulose as an alternative fat replacer in a model food system. Innov. Food Sci. Emerg. Technol..

[137] Amoli P.I., Hadidi M., Hasiri Z., Rouhafza A., Jelyani A.Z., Hadian Z., Khaneghah A.M., Lorenzo J.M. (2021). Incorporation of low molecular weight chitosan in a low-fat beef burger: Assessment of technological quality and oxidative stability. Foods.

[138] Cruz-Romero M.C., O’Flynn C.C., Troy D., Mullen A.M., Kerry J.P. (2021). The use of potassium chloride and tapioca starch to enhance the flavour and texture of phosphate- and sodium-reduced low fat breakfast sausages manufactured using high pressure-treated meat. Foods.

[139] Santos J.M.D., Ignácio E.O., Bis-Souza C.V., Silva-Barretto A.C.D. (2021). Performance of reduced fat-reduced salt fermented sausage with added microcrystalline cellulose, resistant starch and oat fiber using the simplex design. Meat Sci..

[140] Ruan C., Zhang Y., Sun Y., Gao X., Xiong G., Liang J. (2019). Effect of sodium alginate and carboxymethyl cellulose edible coating with epigallocatechin gallate on quality and shelf life of fresh pork. Int. J. Biol. Macromol..

[141] Cao S., Wang S., Wang Q., Lin G., Niu B., Guo R., Yan H., Wang H. (2023). Sodium alginate/chitosan-based intelligent bilayer film with antimicrobial activity for pork preservation and freshness monitoring. Food Control.

[142] Xue T., Jiang Q., Xiang L., Xiao J., Fan D., Wang M., Zhao Y. (2023). Effect of chemical modification of κ-carrageenan on its inhibitory effect against heterocyclic amine (HAs) formation in roasted tilapia fish patties. Int. J. Biol. Macromol..

